# Peptide Derivatives of the Zonulin Inhibitor Larazotide (AT1001) as Potential Anti SARS-CoV-2: Molecular Modelling, Synthesis and Bioactivity Evaluation

**DOI:** 10.3390/ijms22179427

**Published:** 2021-08-30

**Authors:** Simone Di Micco, Simona Musella, Marina Sala, Maria C. Scala, Graciela Andrei, Robert Snoeck, Giuseppe Bifulco, Pietro Campiglia, Alessio Fasano

**Affiliations:** 1 European Biomedical Research Institute of Salerno (EBRIS), Via Salvatore de Renzi 50, 84125 Salerno, Italy; s.musella@ebris.eu (S.M.); AFASANO@mgh.harvard.edu (A.F.); 2 Dipartimento di Farmacia, Università degli Studi di Salerno, Via Giovanni Paolo II 132, 84084 Fisciano, Salerno, Italy; msala@unisa.it (M.S.); mscala@unisa.it (M.C.S.); bifulco@unisa.it (G.B.); pcampiglia@unisa.it (P.C.); 3 Department of Microbiology, Immunology and Transplantation, Rega Institute for Medical Research, KU Leuven, 3000 Leuven, Belgium; graciela.andrei@kuleuven.be (G.A.); robert.snoeck@kuleuven.be (R.S.); 4 Mucosal Immunology and Biology Research Center, Massachusetts General Hospital–Harvard Medical School, Boston, MA 02114, USA

**Keywords:** SARS-CoV-2, peptide, FRET, molecular docking, molecular dynamics, MM-GBSA, drug repurposing, antiviral

## Abstract

A novel coronavirus, severe acute respiratory syndrome coronavirus 2 (SARS-CoV-2), has been identified as the pathogen responsible for the outbreak of a severe, rapidly developing pneumonia (Coronavirus disease 2019, COVID-19). The virus enzyme, called 3CLpro or main protease (M^pro^), is essential for viral replication, making it a most promising target for antiviral drug development. Recently, we adopted the drug repurposing as appropriate strategy to give fast response to global COVID-19 epidemic, by demonstrating that the zonulin octapeptide inhibitor AT1001 (Larazotide acetate) binds M^pro^ catalytic domain. Thus, in the present study we tried to investigate the antiviral activity of AT1001, along with five derivatives, by cell-based assays. Our results provide with the identification of AT1001 peptide molecular framework for lead optimization step to develop new generations of antiviral agents of SARS-CoV-2 with an improved biological activity, expanding the chance for success in clinical trials.

## 1. Introduction

The recent outbreak of COVID-19 pandemic, caused by severe acute respiratory syndrome-Coronavirus-2 (SARS-CoV-2), has raised serious global concern for public health. Due to the highly contagious nature of this life-threatening virus, new therapeutics are urgently required to counteract its transmission [1]. SARS-CoV-2 is a medium-sized, enveloped, positive-strand RNA virus (~30 kb) of genus *Betacoronavirus*, consisting of 29,903 nucleotides, flanked by two untranslated sequences of 254 and 229 nucleotides at the 5′-and 3′-ends, respectively [2,3]. The viral genome has 12 protein-coding regions, which deciphers two categories of proteins: several structural proteins conferring characteristic global shape to the virus and participating to viral entry in the host, and non-structural proteins which assist virion in the infection and replication by conserving a linear arrangement [4]. SARS-CoV-2 enters human cells through the binding to the angiotensin-converting enzyme 2 (ACE2) by its viral spike protein. The spike protein S forms the outer layer of the coronavirus, giving the characteristic crown-like aspect, and initiates host cell invasion [5]. The next step is the replication process, through the transcription of its RNA viral genome. A typical CoV genome contains at least six ORFs (*Open Reading Frame*) which usually encode four well-conserved and characterized structural proteins: S (spike), E (envelope), M (membrane) and N (nucleocapsid) proteins. Precisely, in all coronaviruses the replication phase is initiated by production of the replicase proteins with the translation of ORF1a and ORF1ab via a –1 ribosomal frame-shifting mechanism [6]. This mechanism produces two large viral polyproteins, pp1a and pp1ab (~450 and ~790 kDa, respectively), which are then cleaved into 16 non-structural proteins (Nsp1–Nsp16), required for correct viral replication and transcription [7,8]. The proteolytic process needed for the formation of the Nsps is mainly conducted by two cysteine proteases: papain-pike protease (PL^pro^), which performs three cleavages, and main protease (M^pro^), also called chymotrypsin-like protease (3CLpro), which is responsible for the remaining 11 cuts [8]. Therefore, processing of the viral polyproteins is required to generate the viral non-structural proteins involved in the formation of the replicase complex, which is responsible for properly structuring virions [9]. M^pro^ protease activity becomes thus crucial for viral life cycle and its inhibition can prevent the virus replication. Accordingly, the vital role of M^pro^ for SARS-CoV-2 replication and the lack of homologous human proteins make it the most promising target for antiviral drug development. M^pro^ digests the polyprotein 1ab at multiple cleavage sites by hydrolysis of the Gln-Ser peptide bond mainly in the Leu-Gln-Ser-Ala-Gly recognition sequence [10], leading to the formation of non-structural proteins (NSPs). This cleavage site in the substrate is distinct from the peptide sequence recognized by human homologs, decreasing the possibility to interfere with off-targets in the host.

**Figure 1:**
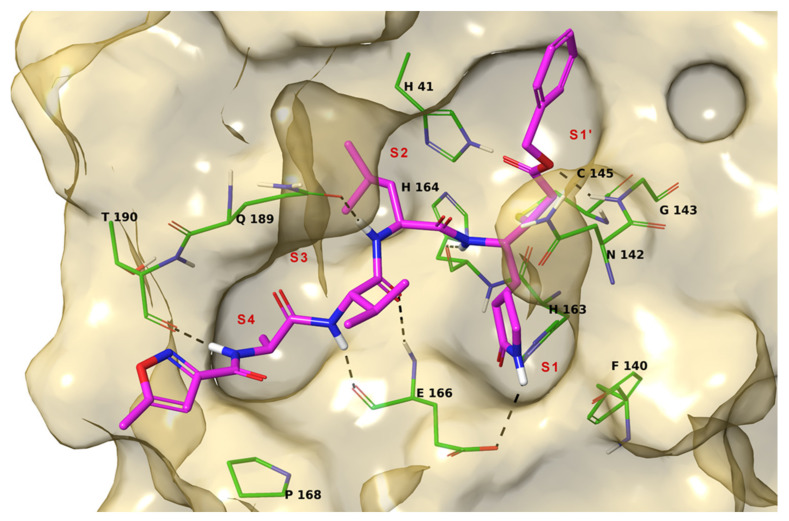
3D model of the interaction between N3 and M^pro^. The protein is represented by gold molecular surface and green tubes (atom color code: C, as tubes; polar H, sky blue; N, dark blue; O, red; S, yellow). N3 is depicted by magenta tubes (atom color code as for protein). The protein sub-pockets are indicated by red labels. The black dashed lines indicate ligand-protein H-bonds.

X-ray crystallography investigations of M^pro^ reveal that the active form of the protein is a homodimer, containing two protomers. Each protomer is formed by a tertiary structure that consists of the catalytic domains I (residue 8–101) and II (residue 102–184) folded into a six-stranded β-barrel hosting the active site, and the helical domain III (residue 201–303) consisting of a cluster of five antiparallel α-helices functionals for the dimerization of the protease [11]. A flexible loop connects domain II to domain III. The M^pro^ active site contains a Cys-His catalytic dyad located in the cleft between domains I and II, in which the side chain of cysteine 145 functions as the nucleophile in the proteolytic process. The active site is composed by canonical sub-pockets that are denoted S1, S1′, S2, S3, and S4 (Figure 1) [12].

The amino acid sequence of the active site is highly conserved among coronaviruses. The catalytic dyad residues are H41 and C145, and residues involved in the binding of substrates include F140, H163, M165, E166, and Q189 [10]. These residues have been found to interact with the ligands co-crystallized with M^pro^ in different studies, like N3 compound that binds irreversibly M^pro^ pocket [13]. Crystallographic data also suggest that Ser1 of one protomer interacts with Phe140 and Glu166 of the other as the result of dimerization. These interactions stabilize the S1 binding pocket; thereby, dimerization of the main protease is likely responsible for its catalytic activity [10].

Recently, we adopted the drug repurposing approach [14] as suitable strategy to give fast response to global COVID-19 epidemic, by demonstrating that the zonulin octapeptide inhibitor AT1001 (Larazotide acetate) [15], currently in phase 3 trials in celiac disease, binds M^pro^ catalytic domain by means of an integrated approach of molecular modeling and fluorescence resonance energy transfer (FRET) assay [16]. Specifically, we observed that AT1001 shares a similar structural pattern to the peptidomimetic inhibitors of that enzyme, N3. These structural motifs, mainly represented by AVL residues in N3 and GVL in AT1001, provided the rationale to investigate AT1001 as a potential new inhibitor of M^pro^ enzyme. Our in silico analysis suggested that the octapeptide docks well in the catalytic domain of M^pro^, presenting a global turn arrangement. In addition, there have been two in-vivo studies establishing the efficacy of AT1001 in mitigating ALI [15]. One of two shows the efficacy of AT1001 therapy during a lethal influenza, suggesting that the protective effect of the drug during influenza infection consists in acute lung injury (ALI) attenuation by diminishing pulmonary edema. To take advantage by previous studies that showed a strong safety profile in both AT1001 administration, systemically (IV) or locally (mucosal airways), we have proceeded in our investigation. The obtained results prompted us to consider AT1001 a new lead compound to a challenging development of potential protease inhibitor candidates. Thus, in the present study we have investigated the putative antiviral activity of AT1001 along with five derivatives, endowed with cap groups and different sequence length.

## 2. Results

Based on our previously reported results [16], we tried to investigate the antiviral activity of AT1001 (**1**, Table 1) by cell-based assays. Considering the intrinsic low membrane permeability of peptides, we designed AT1001 derivatives endowed of cap groups (**2**–**6**, Table 1) in order to mask the charged free N-and C-terminal positions of **1** at physiological pH to improve the cellular wall crossing as theoretically predicted (Table S1) [17]. In particular, we inserted small cap groups, such as acetyl and pivaloyl, at the N-terminus in order to preserve the main interactions given by the parent compound accordingly to the chemical modifications without any steric hindrances [16], obtaining: **2** (Ac-GGVLVQPG-NH_2_), **3** (Ac-GGVLVQPG-NHCH_3_), **4** (Piv-GGVLVQPG-NHCH_3_). Furthermore, by maintaining a cap group on the N-terminus, a C-terminus methyl amide group was introduced to give peptide **3** and **4.** This kind of modification can increase lipophilicity and reduce the capability to form hydrogen bonds, thus facilitating the penetration of peptides across biological membranes and improving pharmacokinetic properties.

Finally, we also considered reducing the sequence length of peptide **2**–**4** to enhance the membrane passage designing the peptides **5** (Ac-GVLVQ-NHCH_3_) and **6** (Ac-GVLV-NHCH_3_). The rational design of shorter analogues was based on molecular dynamics investigation, integrated by MM-GBSA predictions, of AT1001 bound to M^pro^ showing that the N- and C-terminal residues (G1, Q6, P7 and G8) fluctuated largely than the remaining amino acids [16].

Following the same in silico investigation of AT1001 [16], we verified that the designed chemical modifications did not affect the global conformation of **2**–**6** in respect to the parent compound (Table 1 and Figure S1). Indeed, we observed small RMSD (Root Mean Square Deviation) values of **2**–**6** binding poses in respect to the parent peptide, ranging in 0.614–1.081 Å (Table 1). Moreover, the analysis of dihedral angles (Table S2) suggested that **2**–**6** preserve a global turn conformation as previously observed for **1** [16].

**Figure 2:**
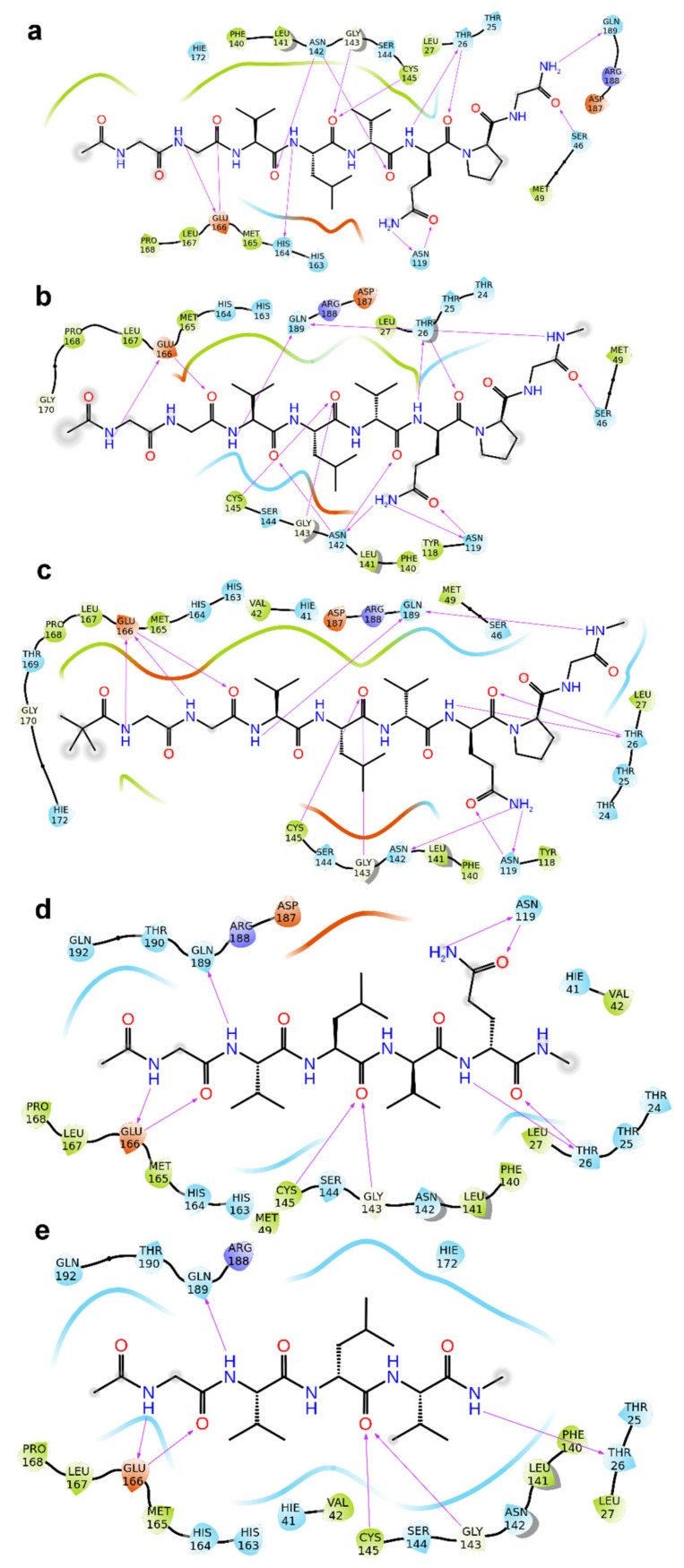
2D panel representing interactions formed by fragments **2** (**a**), **3** (**b**), **4** (**c**), **5** (**d**) and **6** (**e**) with M^pro^. In each panel (**a**–**e**) the molecular structure of **2**–**6** is depicted in black, while the protein amino acids are indicated with three letter code, encircled with colored lines (negatively charged amino acids, red; positively charged amino acids, dark blue; polar amino acids, light blue; hydrophobic amino acids, green circles). The purple arrows indicate H-bonds. The arrows are directed from donor to acceptor of H-bond.

Furthermore, **2**–**6** kept most of the interactions observed for **1** with macromolecular counterparts (Figure 1, Figure 2 and Figure S1). In detail, the V3 is accommodated in S2 pocket delimited by H41, M49, M165, D187, Q189, and its backbone NH is H-bonded to Q189 side chain. The L4 accepts two H-bonds from main chain NH of G143 and C145 and gives van der Waals contacts with N142, H163, E166, F140, L141, H172 of S1 pocket. It is noteworthy that the van der Waals interactions identified for V3 and L4 of **1–6** are observed in the co-crystal structure with N3 (by its leucine and 3-methylpyrrolidin-2-one group) and calpeptin (by its leucine and butyl group) [18]. The G1 and G2 NH group are engaged in H-bond with a backbone of E166, and the CO group of G2 accepts an H-bond from NH of E166. The Q6 side chain is hydrogen bonded to the side chain of N119. The backbone CO of Q6 and P7 accept an H-bond from NH group of T26 and N142 side chain, respectively. The C-terminal carboxylate is H-bonded to side chains of S46 and Q189. The latter interactions were not hampered accordingly to the chemical substitution of carboxylic acid into amide group of **2** (Figure 2a and Figure S1a). As expected, the NHCH_3_ group of **3** and **4** preserves the hydrogen bond but at longer acceptor-donor distance (~0.6 Å) compared to the amide group of **2**. The acyl group at N-terminal also contribute with van der Waals contacts, and **3** and **4** are hydrogen bonded to side chain of Glu166 by their NH group of G1. For shorter peptides (**5** and **6**), the acetyl group is accommodated into S3 pocket formed by M165, L167, P168 and T190. Compared to N3 and calpeptin, **2**–**6** establish further van der Waals contacts with another deep crevice delimited by T25, T26, L27, H41, M49, and C145.

We monitored, over time, the crucial ligand-protein contacts identified for **2**–**6** docked poses into M^pro^ catalytic cavity by means of molecular dynamics simulations (100 ns, 310 K). The inspection of trajectories revealed that **2**–**6** keep most of the interactions with M^pro^ surrounding macromolecular residues for the duration of the whole simulations (>50%), especially with: T26, N119, N142, G143, C145, Hi164, E166, Q189 (Figure S2). The heavy-atom-positional RMSD (root mean square deviations) of **2**–**6**, referenced to protein main chain (Figures S3–S7) appears constant through the trajectory, and their atom-relative orientation is kept over time, confirming the RMSD values of **2**–**6** docked poses compared to AT1001. Moreover, unfavorable conformational rearrangement of the enzyme was not observed during trajectories (Figure S8). We evaluated the trend of predicted MM-GBSA ΔG_bind_ by calculations from molecular dynamics of each ligand-protein. The analysis of the averaged binding energies (Table S3) revealed the agreement with the values obtained from docked complexes, further corroborating their stability. Furthermore, the breakdown of the averaged MM-GBSA binding free energy of residues surrounding **2**–**6** at a distance of 5 Å (Table S4) agree with interactions observed from molecular docking and molecular dynamics investigations.

**Scheme 1:**
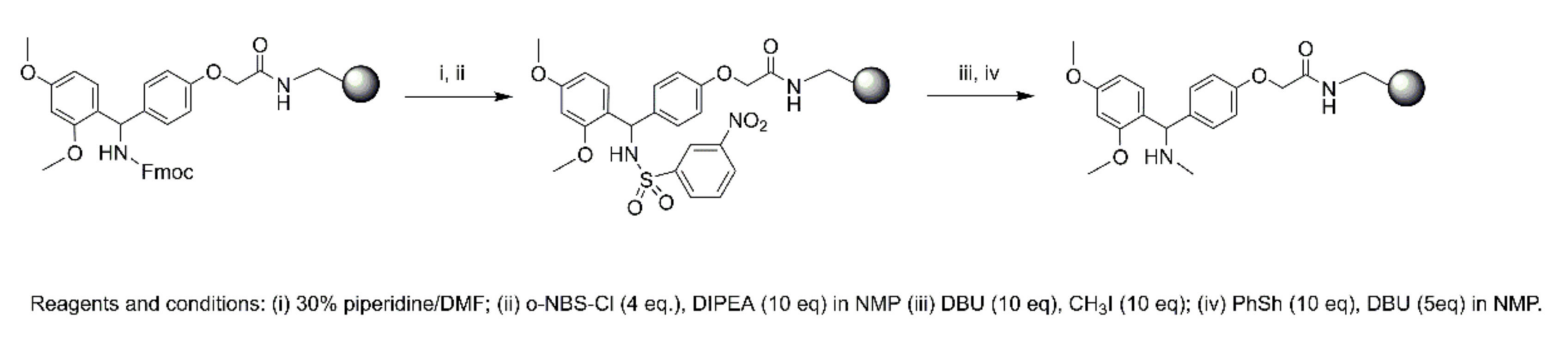
On resin N-methylation.

The compounds (**1**–**6**) were synthesized according to the microwave-assisted peptide synthesis using standard Fmoc methodology. In this work, the synthesis of this type of modified peptide, was performed on a Rink Amide-resin. The whole reaction was carried out following Scheme 1. The Rink amide Fmoc group was first removed using piperidine in DMF to give the free amine, which was then reacted with o-NBS-Cl and DIEA in NMP. The newly formed sulfonamide was deprotonated using DBU in NMP [19] and the resulting anion then reacted with methyl iodide. Next, the complete deprotection of o-NBS group was performed using DBU, as bases, and thiophenol, as thiols (Scheme 1, see also material and methods for further details).

Before cell-based assay aimed to evaluate the antiviral activity, we checked the binding of AT1001 analogues by FRET assay. As previously kinetic measurements of enzymatic of AT1001 against M^pro^ showed a shape-bell dose-response profile with a maximum inhibitory activity at 27% at 0.1 μM, we checked if **2**–**6** maintained the inhibitory activity profile of the parent compound (Table 1). As expected, we observed a similar percentage of activity of AT1001 (Table 1).

Based on these results we proceeded with antiviral tests (Table 1). The different peptides were evaluated for their efficacy in inhibiting the replication of two SARS-CoV-2 clinical strains (UC-1074 = 1.58 × 10^4^ CCID_50_/mL and UC-1075 = 1.08 × 10^6^ CCID_50_/mL) in Vero cells with remdesivir included as reference compound. The peptides **1**, **3** and **5** a negligible activity against SARS-CoV-2; only peptide **2** against one of the SARS-CoV-2 strain (UC-1074) showed a micromolar range activity (EC_50_ = 17.6 ± 2.4 µM, Table 1), similar to the reported value of N3 (EC_50_ = 16.77 ± 1.70 µM) [12] but lower then calpeptin (EC_50_ = 0.072) [18] and remdesivir (Table 1). However, the remaining peptides (**4** and **6**) presented a comparable activity respect to **2**, even though they were lower. As expected, increasing the virus titer on Vero cells, there is a decrease in antiviral activity by tested compounds. All peptides altered cell morphology at a concentration of 100 µM and inhibited Vero cell growth with CC_50_ values in the range of 74–83 µM, comparable to the reference compound remdesivir.

We also evaluated the **1**–**6** antiviral activity against two unrelated viruses: cytomegalovirus (CMV, Table 2) and varicella-zoster virus (VZV, Table 3). In order to measure peptides effectiveness against cytomegalovirus, we used Ganciclovir and Cidofovir as known antiviral compounds; while we choose Aciclovir and Birivudine as specific reference compounds against VZV virus.

Indeed, no activity for **1**–**6** was detected by treated the infected cells with cytomegalovirus, whereas against varicella-zoster virus was observed an activity in high micromolar range.

## 3. Discussion

Recently, we repurposed the zonulin octapeptide inhibitor AT1001 for the SARS-CoV-2 treatment. Our structural studies were limited to in silico analysis integrated by experiments demonstrating the inhibition of main protease, indicating a potential antiviral activity. Based on the previous results we investigated the anti-SARS-CoV-2 activity of AT1001, along with five derivatives. The latter are structurally featured with capped N-and C-terminals, considering the intrinsic low cell membrane permeability of peptides. AT1001 is currently in Phase 3 trials in celiac patients, showing a strong safety beyond a great efficacy for this indication. Thus, in order to remain consistent with structure of AT1001 and preserve its robust safety profile, we introduce small cap groups. The theoretical investigation integrated by FRET assay suggested that the chemical modifications did not affect the binding towards the virus enzyme. The biological activity investigation showed that **2**, followed by peptides **4** and **6**, gives a comparable antiviral activity respect to N3, and could be considered for the hit to lead optimization step. The better antiviral activity could be ascribed by an improved membrane permeability as theoretically predicted, beyond preserving the affinity towards the biological target. Moreover, the experimental results are in qualitative agreement with in silico analysis. Indeed, the peptide **2**, endowed with an amide at C-terminal, established more favorable H-bond with S46 and Q189 respect to **3** and **4** featured of NHCH_3_ group in that position. We envisaged to increase the membrane crossing by shortening the sequence length of parent compound, by designing, synthesizing and testing the derivatives **5** and **6**. The shorter peptide **6** maintained the antiviral activity in the range of **2**, even though establishing less extended interactions with macromolecular counterparts gives rise to a lower biological activity than **2**. Compared to **5**, peptide **6** presents the right balance between affinity and cell membrane permeability. Indeed, the lower activity of **5** compared to **6**, could be ascribable to the lower cell permeability. It is worth of note that the peptides were incubated for five days in cells. Even though this long time could be detrimental for the activity due to possible peptidase hydrolysis, we observed an antiviral action against infected cells suggesting an improvement of antiviral profile in shorter incubation times.

It should be highlighted that for anti-cytomegalovirus and anti-varicella-zoster virus tests, we used the standard HEL cells, instead of Vero cells currently employed for anti-SARS-CoV-2 experiments. Thus, these data could not directly address a selectivity property by our tested compounds against SARS-CoV-2. However, the toxicology profiles obtained on Vero and human embryonic lung cells demonstrated strong safety by using the tested compounds.

Recently, the M^pro^ inhibitor PF-07321332 was announced by Pfizer (https://cen.acs.org/acs-news/acs-meeting-news/Pfizer-unveils-oral-SARS-CoV/99/i13/, accessed date: 15 July 2021) as promising Phase 1 clinical candidate. To date, in our opinion, our results could not be easily compared with PF-07321332 inhibitor. Firstly, peptides **1**–**6** are non-covalent M^pro^ inhibitors, despite the Pfizer candidate acting as covalent M^pro^ binder. Furthermore, our identified lead compounds require pharmacokinetics and pharmacodynamics improvements. However, the lack of an approved COVID-19 therapy still require drug discovery campaign and we do believe that by expanding the chemical diversity of the potential ligands, increases the possibility to reach more efficacious and safer treatment. To the best of our knowledge, our proposed leads represent the first class of peptide inhibitors of M^pro^. Despite reported known M^pro^ binders so far, our structural investigation suggest that tested ligands interact with another deep subpocket (delimited by T25, T26, L27, H41, M49 and C145), giving new insights for drug design. In addition, our main goal remains the attempt to discover new drugs with a remarkable antiviral activity against SARS-CoV-2 maintaining a low cytotoxic profile that characterizes many drugs used, like Remdesivir. In fact, although remdesivir is currently approved by the USA-FDA to treat COVID-19 patients, its clinical efficacy remains debatable. Considering the strong safety profile of AT1001 administered to human subjects, it represents a good starting point to circumvent the adverse effect showed by most of repurposed drugs.

Overall, the present data has strongly suggested the identification of AT1001 peptide molecular framework for the hit to lead optimization step to develop new generations of antiviral agents for the treatment of SARS-CoV-2. Furthermore, the structural information and biological activities observed lay foundations for an ongoing rational design of both peptides and peptidomimetics with improved pharmacodynamics and pharmacokinetics properties. These outcomes could provide further chances of disclosing an interesting hit to be directed towards further investigations and for adding another piece to tackle the hard challenge to develop clinical candidates against SARS-CoV-2, which lacks any therapeutical treatment so far.

## 4. Materials and Methods

### 4.1. Molecular Docking

The Build Panel of Maestro (version 11, Schrödinger, LLC., New York, NY, USA) was used to construct the 3D structures of **2**–**6**, and successively optimizing their geometries through: OPLS3 force field [20], Polak-Ribière conjugate gradient algorithm (maximum derivative <0.001 kcal/mol), GB/SA (generalized Born/surface area) [21] solvent treatment of H_2_O. Then, the peptides were processed by LigPrep [22], accounting for the protonation states at pH of 7.0 ± 1.0. Protein Preparation Wizard [23,24] was employed to process the X-ray structure of M^pro^ (PDB ID: 6LU7) [12]: bond order assignment and hydrogen addition; missing side chain and loop check; check of alternate positions of the residues, side chain charge assignment at pH 7.0 ± 1.0; H-bond network improvement through the optimize preference. The H_2_O molecules were removed. Molecular docking predictions were carried out by Glide (v. 7.2, Schrödinger, LLC., New York, NY, USA), by using peptide specific protocol (SP-PEP) [25]. The docking protocol was validated by redocking the co-crystallized N3 with M^pro^ and overlapping the docked and experimental poses (Figure S9; RMSD = 1.076 Å). The receptor grid, proper for peptide docking, was sized as 10 Å inner and 22 Å outer boxes, with a center coordinates: −10.80 (x), 12.53 (y), 68.70 (z). A first set of SP-PEP was carried out by means of default parameters, with extended sampling option for conformer generation and expanded sampling for initial pose selection. 100 poses for **2**–**6** were generated, treating the ligands as flexible allowing only trans conformation for amide bonds. The following scoring contribution were considered: Epik state penalty; reward of intramolecular H-bonds; and aromatic hydrogen. The **2**–**6** docked poses obtained from the first calculation run were used as starting conformations for a second round by means of the same parameters generating 100 conformations for each input one. All 10,100 conformations from the first two calculation sets were collected and classified by docking score, and the best 100 ranked conformers were used as input for a third round of predictions generating further 10,000 docked poses. Predicted apparent Caco-2 cell permeability (QPPCaco) was calculated by QuikProp of Schrödinger suite [17], using default parameters and Caco-2 cells as model.

Maestro (version 11, Schrödinger, LLC., New York, NY, USA) was utilized for theoretic study and to generate all depictions.

### 4.2. MM-GBSA

The best 100 ranked conformers of **2**–**6** from the three rounds of molecular docking calculations were rescored by MM-GBSA predictions, by means of the Prime 3.1 [26,27] module of the Schrödinger suite (Schrödinger, LLC., New York, NY, USA) applying default parameters. A distance of 5 Å from each ligand was used to define flexible residues. Briefly, the binding energies of the protein and ligand are calculated by: Prime Energy + Implicit Solvent Energy in the free and bound states, accounting for the prediction of the energetic penalty due to strain between the ligand and protein in both states.

The φ, ψ and χ1 angles of best docked pose of each ligand were analyzed by PROMOTIF 3.0 (School of Animal and Microbial Sciences University of Reading, Whiteknights, UK; Biomolecular Structure and Modelling Unit, Department of Biochemistry and Molecular Biology, University College, Gower Street, London, UK) [28].

### 4.3. Molecular Dynamics

The docked complexes of **2**–**6** bound to M^pro^ were used for molecular dynamics simulation. These complexes were prepared by System Builder (Schrödinger, LLC., New York, NY, USA) [29] in Desmond v. 4.9 (DE Shaw Research, New York, NY, USA) [30,31], by suing: a cubic box with a 10 Å buffer distance, OPLS3 force field [20], the TIP3P [32] solvation model, Na^+^ and Cl^−^ ions for electroneutrality, along with a NaCl solution (0.15 M). These systems were firstly optimized by the LBFGS methodology using default parameters and then underwent to the following relaxation protocol: (1) restrained solute heavy atom NVT simulation (2 ns, 10 K, small time steps); (2) restrained solute heavy atom NVT simulation (240 ps, 10 K with Berendsen thermostat, fast temperature relaxation constant) 1 ps of velocity resampling; (3) restrained solute heavy atom NPT simulation (240 ps, 10 K) with Berendsen thermostat and Berendsen barostat (1 atm), fast temperature relaxation constant, slow pressure relaxation constant, velocity resampling of 1 ps; (4) restrained solute heavy atom NPT ensemble simulation (240 ps) through Berendsen barostat (1 atm) and Berendsen thermostat (310 K), fast temperature relaxation constant, slow pressure relaxation constant, velocity resampling of 1 ps; (5) 480 ps NPT simulation employing Berendsen thermostat (310 K) and Berendsen barostat (1 atm), normal pressure relaxation constant and fast temperature relaxation constant. Unrestrained molecular dynamics of 100 ns (310 K) with NPT (1.01 bar) ensemble class were run, through 1.2 ps of recording time and 2.0 fs of integration time step. Each equilibration phase was evaluated by the Simulation Quality Analysis tool of Desmond, examining pressure, volume, temperature, total and potential energies.

### 4.4. Synthesis

#### 4.4.1. Material

N^α^-Fmoc-protected amino acids, coupling reagents (HOAt, HBTU), Fmoc-L-Gly-Wang resin, Rink Amide-resin, N, N-Diisopropylethylamine (DIEA), piperidine and trifluoroacetic acid (TFA) were purchased from Iris Biotech (Marktredwitz, Germany). Peptide synthesis solvents, reagents, as well as CH_3_CN for High Performance Liquid Chromatography (HPLC) were reagent grade and were acquired from commercial sources and used without further purification unless otherwise noted.

#### 4.4.2. Microwave Peptide Synthesis

The synthesis of peptides (**1**–**6**) was performed with a solid phase approach using a standard Fmoc methodology on a Biotage Initiator + Alstra automated microwave synthesizer (Biotage, Uppsala, Sweden).

##### Synthesis of **1** 

Peptide was synthesized on an Fmoc-L-Gly-Wang resin (0.7 mmol/g, 150 mg), previously deprotected with 30% piperidine/DMF (1 × 3 min, 1 × 10 min) at room temperature. The resin was then washed with DMF (4 × 4.5 mL) and protected amino acids added on to the resin stepwise. Coupling reactions were performed using Nα-Fmoc amino acids (4.0 eq., 0.5 M), HBTU (3 eq, 0.6 M), HOAt (3 eq, 0.5 M), and DIEA (6 eq, 2 M) in N-methyl-2-pyrrolidone (NMP) for 10 min at 75 °C (2×). After each coupling step, the Fmoc protecting group was removed as described above. The resin was washed with DMF (4 × 4.5 mL) after each coupling and deprotection step. The N-terminal Fmoc group was removed, the resin was washed with DCM (7×), and the peptide released from the resin with TFA/TIS/H2O (ratio 90:5:5) for 3 h. The resin was removed by filtration and the crude peptide recovered by precipitation with cold anhydrous ethyl ether to give a white powder that was then lyophilized.

##### Synthesis of **2** 

Peptide was synthesized onto a Rink Amide-resin (150 mg, loading 0.71 mmol/g), previously deprotected with 30% piperidine/DMF (1 × 3 min, 1 × 10 min) at room temperature. The synthesis was then performed using the conditions stated for **1**.

##### Synthesis of **3**–**6** 

Peptides were synthesized onto a Rink Amide-resin (150 mg, loading 0.71 mmol/g), previously deprotected with 30% piperidine/DMF (1 × 3 min, 1 × 10 min) at room temperature. The resin was then washed with DMF (4 × 4.5 mL) and the primary amino group was protected and simultaneously activated by reacting it with o-NBS-Cl (4 eq) and DIEA (10 eq) in N-methyl-2-pyrrolidone (NMP) overnight. After this step, the methylation was carried out by treating the resin with DBU (10 eq, 30 min) and CH_3_I (10 eq, 30 min) in NMP (2×). Then, the o-NBS protecting group was removed with thiophenol (10 eq) and DBU (5 eq) for 30 min. All steps were performed at room temperature. The synthesis was then performed as described above.

##### N-Terminal Capping

A capping step was performed: for peptides **2**, **3**, **5** and **6** by adding a solution of Ac_2_O/DCM (1:3) shaking for 30 min and for **4** by adding a solution of Piv-Cl (4 eq.) e DIPEA (8 eq.) in DCM for 30 min.

#### 4.4.3. Purification and Characterization

All crude peptides were purified by RP-HPLC on a preparative C18-bonded silica column (Phenomenex Kinetex AXIA 100 Å, 100 × 21.2 mm, 5 µm) using a Shimadzu SPD 20 A UV/VIS detector, with detection at 214 and 254 nm (Table S5). Mobile phase was: (A) H_2_O and (B) ACN, both acidified with 0.1% TFA (*v*/*v*). Injection volume was 5000 µL; flow rate was set to 17 mL/min. The following gradient was employed: 0–18 min, 5–50% B, 18.01–20 min, 50–90% B, 20.01–21 min, returning to 5% B. Analytical purity and retention time (tr) of each peptide were determined using HPLC conditions in the above solvent system (solvents A and B) programmed at a flow rate of 0.500 mL/min, fitted with analytical C-18 column (Phenomenex, Aeris XB-C18 column, 100 mm × 2.1, 3.6 μm). LC gradient was the following: 0–7 min, 5–90% B, 7.01–8 min, returning to 5% B, 8–11 min, isocratic for 3 min. All analogues showed >99% purity when monitored at 220 nm. Homogeneous fractions, as established using analytical HPLC, were pooled and lyophilized.

Ultra high resolution mass spectra were obtained by positive ESI infusion on a LTQ Orbitrap XL mass spectrometer (Thermo Scientific, Dreieich, Germany), equipped with the Xcalibur software for processing the data acquired. The sample was dissolved in a mixture of water and methanol (50/50) and injected directly into the electrospray source, using a syringe pump, at constant flow (15 µL/min). Analytical data are shown in Supplementary Materials (Table S5 and Figures S10–S15).

### 4.5. Enzymatic Inhibition Assays

The recombinant SARS-CoV-2 M^pro^ (Proteros) (20 nM at a final concentration) was mixed with serial dilutions of AT1001 and Dabcyl-KTSAVLQSGFRKM-E(Edans)-NH_2_ substrate (5 μM) in 20 μL (reaction volume) assay buffer solution (20 mM HEPES, pH 7.5, 1 mM DTT, 1 mM EDTA, 100 mM NaCl, 0.01% Tween20). The appropriate volume of substrate was added in reaction buffer along with 42.5 nL compound in 100% DMSO. Finally, the appropriate volume of target enzyme was added, and the reaction started with an incubation time of 10 min. The fluorescence signal of the Edans was monitored at an emission wavelength of 500 nm by exciting at 360 nm, by means of Pherastar FSX microplate Reader (BMG LABTECH GmbH, Ortenberg, Germany). Calpeptin was used as reference to set up the experiments.

### 4.6. Biological Activity

#### 4.6.1. SARS-CoV-2

Vero cells (ATCC-CCL81^TM^) were used to evaluate the activity of the peptides against SARS-CoV-2. Cells were grown in Dulbecco’s Modified Eagle’s Medium (DMEM, ThermoFisher, Merelbeke, Belgium) supplemented with 10% fetal calf serum (FCS), 2 mM L-glutamine, 0.1 mM non-essential amino acids, 1 mM sodium pyruvate and 10 mM HEPES at 37 °C in a 5% CO_2_ humidified atmosphere. Two SARS-CoV-19 strains, denoted UC-1074 and UC-1075, were isolated in Vero cells from nasopharyngeal swabs of two COVID-19 patients who had, respectively, a Ct of 19 and 22, for detection of SARS-CoV-2 E protein by RT-qPCR real-time reverse transcription PCR (RT-qPCR). The infectious virus titer of the clinical isolates was determined in Vero cells and expressed as 50% cell culture infectious dose (CCID_50_) per mL, being of 1.58 × 10^4^ (UC-1074) and 1.08 × 10^6^ (UC-1075) CCID_50_/mL. For the antiviral assays, Vero cells were seeded in 96-well plates at a density of 1 × 104 cells per well in DMEM 10% FCS medium. After 24 h growth, the cell culture medium was removed and cells were treated with different compound concentrations in DMEM 2% FCS and mocked-infected or SARS-CoV-2-infected with 100 CCID_50_/well (final volume 200 µL/well). After 5 days of incubation at 37 °C, viral CPE was recorded microscopically and the 50% effective concentration (EC_50_) was calculated for each peptide (Figure S16) and remdesivir (reference anti-SARS-CoV-2 compound). In parallel, the cytotoxic effects of the derivatives were assessed by evaluating the MCC (minimum cytotoxic concentration that causes a microscopically detectable alteration of cell morphology). The effects of the compounds on cell growth were as well determined by counting the number of cells with a Coulter counter in mock-infected cultures and expressed as cytostatic concentration required to reduce cell growth by 50% (CC_50_). All SARS-CoV-2-related work was conducted in the high-containment BSL3+ facilities of the KU Leuven Rega Institute (3CAPS) under licenses AMV 30,112,018 SBB 219 2018 0892 and AMV 23,102,017 SBB 219 2017 0589 according to institutional guidelines.

#### 4.6.2. Cytomegalovirus and Varicella-Zoster Virus

The compounds were investigated against the following viruses: varicella-zoster virus (VZV) wild-type strain Oka (ATCC VR-795), thymidine kinase deficient (TK−) VZV strain 07−1 (kindly provided by Shiro Shigeta, Fukushima Medical Center, Fukushima, Japan), human cytomegalovirus (HCMV) strains AD-169 (ATCC VR-538) and Davis (VR-807). The antiviral assays are based on the inhibition of virus-induced cytopathic effect (HCMV) or plaque formation (VZV) in human embryonic lung (HEL) fibroblasts (HEL 299 (ATCC^®^ CCL-137™). Confluent cell cultures in microtiter 96-well plates were inoculated with 100 CCID_50_ of virus (1 CCID_50_ being the virus dose to infect 50% of the cell cultures) or with 20 plaque forming units (PFU) (VZV). After adsorption for 2 h, the viral inoculum was removed and the cultures were further incubated in the presence of varying concentrations of the test compounds. Viral cytopathic effect or plaque formation was recorded after 5 (VZV) or 6−7 (CMV) days post-infection. Antiviral activity was expressed as the EC_50_ or compound concentration required inhibiting virus induced cytopathic effect or viral plaque formation by 50% (Figure S16). The cytostatic activity measurements were based on the inhibition of cell growth. HEL cells were S12 seeded into 96-well microtiter plates at a rate of 5 × 10^3^ cells/well and incubated for 24 h. Then, medium containing the test compounds at different concentrations was added. After 3 days of incubation at 37 °C, the cell number was determined using a Coulter counter. The cytostatic concentration was calculated as the CC_50_, or compound concentration required to reduce cell proliferation by 50% relative to the number of cells in the untreated controls. CC_50_ values were estimated from graphic plots of the number of cells (percentage of control) as a function of the concentration of the test compounds. Alternatively, cytotoxicity of the test compounds was expressed as the minimum cytotoxic concentration (MCC) or compound concentration that causes a microscopically detectable alteration of cell morphology.

## Figures and Tables

**Table 1:** Root mead square deviation (RMSD), predicted ΔG_bind_, enzymatic inhibition, and activity against SARS-CoV-2 in Vero cells of **1**–**6**.

Peptide	RMSD (Å)	ΔG_bind_ (kcal/mol)	Inhibition ^a^ (%)	Antiviral Activity (EC_50_, µM) ^b^		Cytotoxicity (µM)	
				UC-1074	UC-1075	Cell Morphology (MCC) ^c^	Cell Growth (CC_50_) ^d^
AT1001 (**1**)	0.000	−106.26	27.7 ± 0.2	>20	>20	100	82.5 ± 6.2
**2**	0.864	−112.34	27.1 ± 0.5	≥17.6 ± 2.4	>20	100	83.0 ± 17.0
**3**	0.664	−109.41	27.4 ± 1.1	>20	>20	100	81.8 ± 2.1
**4**	0.696	−105.79	26.2 ± 0.3	≥20	>20	100	74.9 ± 4.9
**5**	0.614	−95.86	27.0 ± 1.1	>20	>20	100	75.0 ± 2.2
**6**	1.081	−80.38	25.0 ± 0.5	≥20	>20	100	77.4 ± 0.7
Remdesivir	-	-	-	0.89 ± 0.44	1.06 ± 0.44	100	70.6 ± 4.9

^a^ Relative to concentration range: 0.1–1 µM^16^. 50% of inhibition was observed for calpeptin. ^b^ Effective concentration required to reduce virus plaque formation by 50%. Virus input was 100 CID_50_. ^c^ Minimum cytotoxic concentration that causes a microscopically detectable alteration of cell morphology. ^d^ Cytotoxic concentration required to reduce cell growth by 50%.

**Table 2:** Activity of the synthesized peptides against cytomegalovirus (HCMV) in human embryonic lung (HEL) cells.

Peptide	Antiviral Activity (EC_50_, µM) ^a^		Cytotoxicity (µM)	
	AD-169 Strain	Davis Strain	Cell Morphology (MCC) ^b^	Cell Growth (CC_50_) ^c^
AT1001 (**1**)	>20	>20	≥100	ND ^d^
**2**	>20	>20	≥20	ND ^d^
**3**	>20	>100	≥20	ND ^d^
**4**	>20	100	100	ND ^d^
**5**	>20	>100	≥100	ND ^d^
**6**	>4	>20	≥20	ND ^d^
Ganciclovir	2.56 ± 0.23	1.18	≥394	350.23
Cidofovir	1.27 ± 0.18	0.67 ± 0.49	≥317	159.22 ± 76.5

^a^ Effective concentration required to reduce virus cytopathic effect by 50%. Virus input was 100 CCID_50_. ^b^ Minimum cytotoxic concentration that causes a microscopically detectable alteration of cell morphology. ^c^ Cytotoxic concentration required to reduce cell growth by 50%. ^d^ Not determined.

**Table 3:** Activity of the synthesized peptides against varicella-zoster virus (VZV) in human embryonic lung (HEL) cells.

Peptide	Antiviral Activity (EC_50_, µM) ^a^		Cytotoxicity (µM)	
	TK+ VZV Strain OKA	TK-VZV Strain 07–1	Cell Morphology (MCC) ^b^	Cell Growth (CC_50_) ^c^
AT1001 (**1**)	44.14	59.06	≥100	ND ^d^
**2**	50.05	48.42	>100	ND ^d^
**3**	58.09	81.09	>100	ND ^d^
**4**	78.20	>100	≥100	ND ^d^
**5**	59.80	>100	>100	ND ^d^
**6**	>20	>20	100	ND ^d^
Aciclovir	8.39	116 ± 93	>444	>444
Birivudine	0.23 ± 0.04	2.13	>300.3	>300.3

^a^ Effective concentration required to reduce virus plaque formation by 50%. Virus input was 20 plaque forming units (PFU). ^b^ Minimum cytotoxic concentration that causes a microscopically detectable alteration of cell morphology. ^c^ Cytotoxic concentration required to reduce cell growth by 50%. ^d^ Not determined.

## Supplementary Materials

The following are available online at www.mdpi.com/article/10.3390/ijms22179427/s1, Table S1. docking scores and QPPCaco values of **1**–**6**; Table S2. Dihedral angle analysis of **2**–**6**; Table S3. average MM-GBSA ΔG_bind_ from molecular dynamics; Table S4. Averaged MM-GBSA ΔG_bind_ for residues from molecular dynamics; Table S5. Analytical data of peptides **1**–**6**; Figure S1. superimposition of AT1001 (purple) with **2**–**6** into M^pro^; Figure S2. Protein-ligand contact histograms during the simulation; Figure S3–S7. RMSD of **2**–**6**; Figure S8. RMSD of protein Cα atoms bound to **2**–**6**; Figure S9. N3 docked and crystallized pose overlay; Figure S10–S15. HRMS spectra and HPLC chromatograms of peptide **1**–**6** title; Figure S16. Dose-response curves.
