# Supplementary material for: Peptide Derivatives of the Zonulin Inhibitor Larazotide (AT1001) as Potential Anti SARS-CoV-2: Molecular Modelling, Synthesis and Bioactivity Evaluation
Source: Int J Mol Sci. 2021 Aug 30;22(17):9427. doi: 10.3390/ijms22179427 (PMC8431481; doi:10.3390/ijms22179427)
Supplement: Supplementary file 1 [file ijms-22-09427-s001.zip › ijms-1321197-supplementary.pdf]

Supplementary Material

Peptide derivatives of the Zonulin Inhibitor Larazotide (AT1001) as potential anti SARS Cov-2: molecular modelling, synthesis and bioactivity evaluation

Simone Di Micco [1,*], Simona Musella [1], Marina Sala [2], Maria C. Scala [2], Graciela Andrei [3], Robert Snoeck [3], Giuseppe Bifulco [2], Pietro Campiglia [2] and Alessio Fasano [1,4]

[1]European Biomedical Research Institute of Salerno (EBRIS), via Salvatore De Renzi 50, 84125 Salerno, Italy.
[2]Dipartimento di Farmacia, Università degli Studi di Salerno, Via Giovanni Paolo II 132, 84084 Fisciano, Salerno, Italy.
[3]Rega Institute for Medical Research, Department of Microbiology, Immunology, and Transplantation, KU Leuven, Leuven, 3000 Belgium.
[4]Mucosal Immunology and Biology Research Center, Massachusetts General Hospital–Harvard Medical School, Boston, MA 02114, United States.

Contents:

**Table S1**	Docking scores and QPPCaco values of **1-6**	S2
**Table S2**	Dihedral angle analysis of **2-6**	S3
**Table S3**	Averaged ΔG_{bind} from md of ligand-protein complexes	S4
**Table S4**	Averaged ΔG_{bind} (kcal/mol) for residues from md	S5
**Table S5:**	Analytical data of peptides **1-6**	S6
**Figure S1**	Superimposition of AT1001 (purple) with **2-6** into M^{pro}	S7
**Figure S2**	Protein-ligand contact histograms during the simulation	S8
**Figure S3-S7**	RMSD of **2-6**	S9-S13
**Figure S8**	RMSD of protein Cα atoms bound to **2-6**	S14
**Figure S9**	N3 docked and crystallized pose overlay	S15
**Figure S10-S15**	HRMS spectra and HPLC chromatograms of peptide **1-6**	S16-S21
**Figure S16**	Dose-response curves	S22

Table S1. Docking scores and Predicted apparent Caco-2 cell permeability (QPPCaco) by QikProP of Schrödinger suite for ligands.

peptide	docking score (kcal/mol)	QPPCaco[a] (nm/s)
AT1001 (**1**)	−12.574	0.002
**2**	−11.522	0.012
**3**	−11.209	0.050
**4**	−11.240	0.143
**5**	−12.217	0.734
**6**	−9.103	40.709

Table S2. Mean values of φ, ψ and χ1 angles and αC distances relative to the bound conformations **2-6** into M^{pro} catalytic site.

				i+1			i+2			αC distance (Å)	
peptide	β-turn	γ-turn	sequence	φ	ψ	χ1	φ	ψ	χ1	i to i+2	i to i+3
**1**	VIa2	-	V5-G8	-109.2	119.8	-177.8	-77.8	-38.2	30	-	4.9
	-	inverse	V3-V5	-84.1	46.6	-60.3	-	-	-	5.8	-
**2**	VIa2	-	V5-G8	-103.8	123.4	-177.7	-78.6	-35.8	30.5	-	5.2
**3**	IV	-	V5-G8	-92.9	125.3	-78.4	-77.9	-41.5	28.7	-	5.2
**4**	IV	-	V5-G8	-98.8	124.5	-75.4	-75.4	-38.0	27.8	-	5.1
	-	inverse	V3-V5	-89.6	47.7	-85.3	-	-	-	5.7	-
**5**	-	inverse	V2-V4	-78.5	48.3	-55.8	-	-	-	5.8	-
**6**	-	inverse	V2-V4	-85.8	54.7	-68.6	-	-	-	5.7	-

Table S3. Averaged ΔG_{bind} from molecular dynamics of ligand-protein complexes predicted by MM-GBSA.

peptide	ΔG_{bind} (kcal/mol)
AT1001 (**1**)	-109.42
**2**	-113.23
**3**	-120.94
**4**	-118.19
**5**	-105.88
**6**	-85.88

Table S4. Averaged ΔG_{bind} (kcal/mol) for residues around 5 Å from ligand, derived from molecular dynamics of ligand-protein complexes predicted by MM-GBSA.

	**1**	**2**	**3**	**4**	**5**	**6**
THR24	-0.68	-0.65	-0.46	-0.80	-0.15	0.00
THR25	**-4.36**	**-4.74**	**-4.68**	**-4.60**	**-4.11**	-0.51
THR26	**-5.83**	**-4.14**	**-5.78**	**-5.88**	**-4.99**	**-1.36**
LEU27	**-2.15**	**-1.79**	**-1.64**	**-2.02**	**-1.89**	**-1.37**
PRO39	-0.22	-0.13	0.00	-0.03	0.00	0.03
HIS41	**-1.86**	**-1.76**	**-1.70**	**-1.70**	**-1.65**	-0.58
VAL42	-0.30	-0.34	-0.31	-0.40	-0.20	-0.12
CYS44	-0.11	-0.09	-0.10	-0.13	0.03	-0.07
THR45	-0.09	-0.26	-0.35	-0.43	-0.01	0.00
SER46	-0.51	-0.19	**-1.51**	-0.73	-0.06	0.04
MET49	**-1.44**	**-1.14**	**-1.92**	**-1.98**	-0.58	-0.20
TYR54	0.10	0.01	-0.02	0.06	-0.04	0.04
TYR118	-0.42	0.01	0.43	0.13	0.26	0.19
ASN119	**-1.96**	**-1.43**	**-1.38**	**-1.30**	**-1.66**	-0.24
PHE140	-0.72	0.24	0.05	-0.16	0.01	-0.37
LEU141	-0.96	-0.59	**-1.43**	-0.76	-0.63	-0.52
ASN142	**-11.19**	**-7.48**	**-9.21**	**-8.41**	**-7.10**	**-4.17**
GLY143	**-3.87**	**-3.47**	**-2.98**	**-3.24**	**-3.38**	**-2.84**
SER144	**-2.01**	**-1.74**	-0.86	**-1.22**	-0.97	-0.98
CYS145	**-4.31**	**-3.49**	**-3.25**	**-3.89**	**-3.99**	**-3.61**
HIS163	**-1.04**	-0.35	**-1.14**	**-1.15**	**-1.14**	-0.95
HIS164	**-3.78**	**-3.35**	**-1.87**	**-2.60**	**-2.69**	**-2.49**
MET165	**-6.79**	**-6.43**	**-6.14**	**-5.23**	**-6.15**	**-4.24**
GLU166	**-6.50**	**-6.19**	**-6.04**	**-4.82**	**-5.86**	**-4.99**
LEU167	-0.77	**-1.69**	**-1.35**	**-1.91**	-0.81	-0.49
PRO168	-0.40	**-1.99**	**-1.75**	**-2.47**	-0.60	1.18
GLY170	-0.01	0.19	-0.06	-0.26	0.14	0.16
HIS172	-0.62	-0.66	-0.93	-0.42	**-1.12**	-0.94
PHE181	-0.03	-0.06	-0.01	0.00	0.05	0.03
ASP187	0.24	-0.29	0.43	-0.07	-0.01	-0.19
ARG188	-0.39	-0.65	**-1.22**	-0.70	-0.37	-0.56
GLN189	**-3.08**	**-2.99**	**-3.49**	**-3.68**	**-3.87**	**-2.93**

The values in bold are ≤-1 kcal/mol.

Table S5. Analytical data of peptides **1-6**.

**Peptide**	**Sequence**	**HPLC** ***k'*** [a]	**ESI-MS**
**1**	GGVLVQPG	6.23	726.4125
**2**	Ac-GGVLVQPG-NH_2	6.68	767.4402
**3**	Ac-GGVLVQPG-$NHCH_3$	5.17	781.4580
**4**	Piv-GGVLVQPG-$NHCH_3$	6.42	823.5031
**5**	Ac-GVLVQ-$NHCH_3$	7.22	570.3594
**6**	Ac-GVLV-$NHCH_3$	5.33	442.3006

[a] k'=[(peptide retention time-solvent retention time)/solvent retention time].

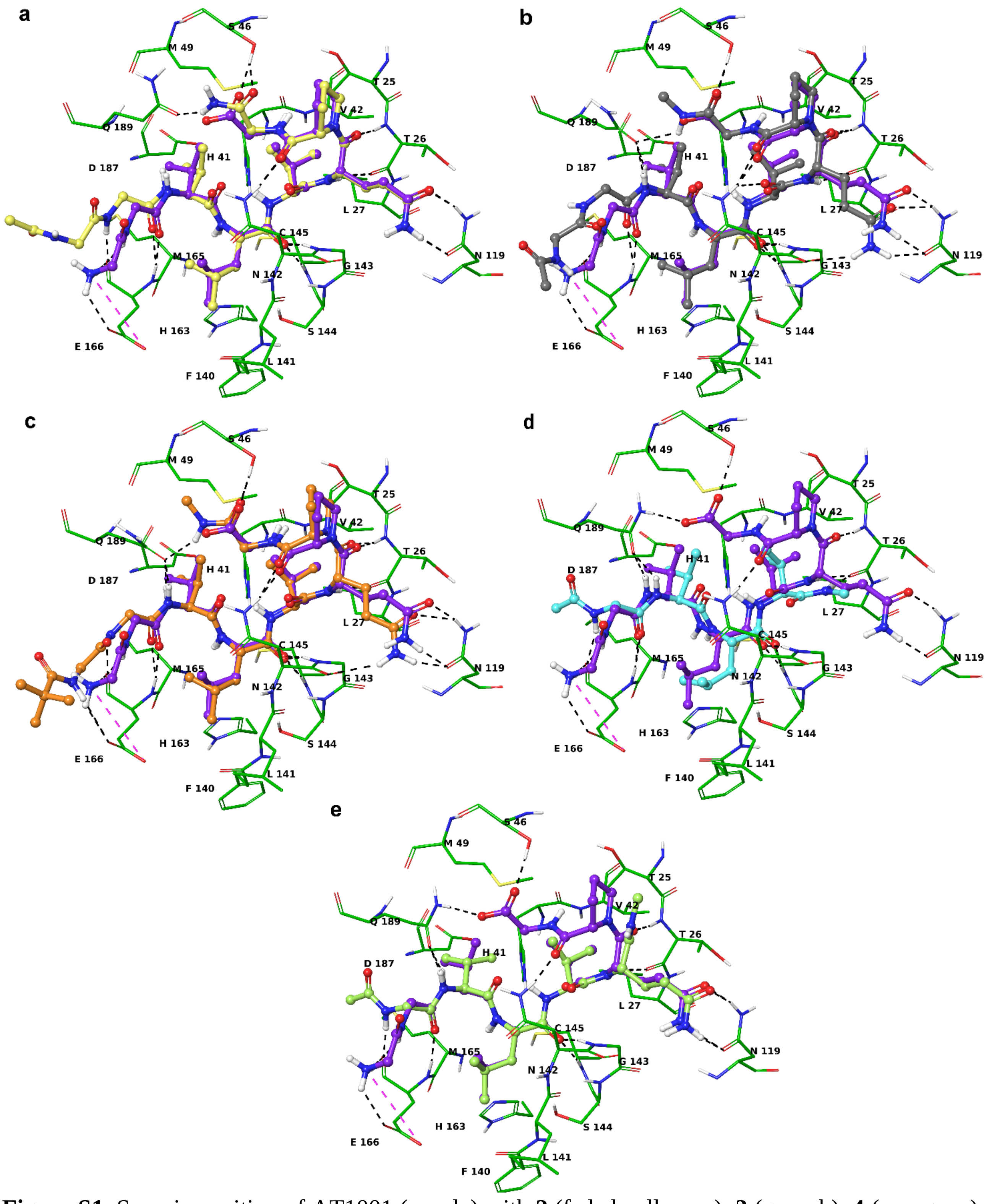

Figure S1. Superimposition of AT1001 (purple) with **2** (faded yellow; a), **3** (grey; b), **4** (orange; c), **5** (faded teal; d), and **6** (faded yellow-green; e), into the catalytic site, necessary for viral transcription and replication.

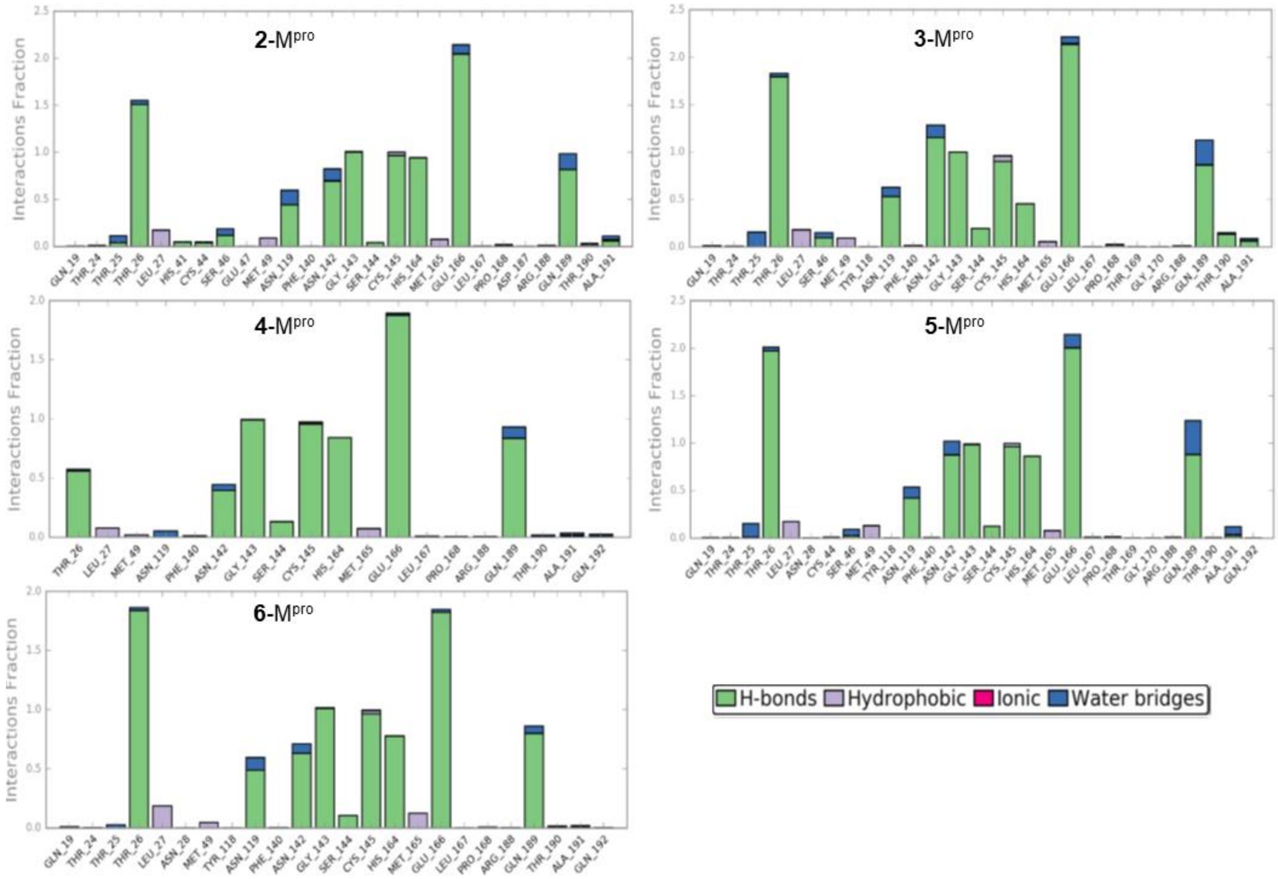

Figure S2. Simulation interaction diagrams. Protein-ligand contact histograms during the simulation. Protein-ligand interactions are categorized into four types: Hydrogen Bonds, Hydrophobic, Ionic and Water Bridges. The stacked bar charts are normalized over the course of the trajectory: for example, a value of 0.7 suggests that 70% of the simulation time the specific interaction is maintained. Values over 1.0 are possible as some protein residue may make multiple contacts of same subtype with the ligand.

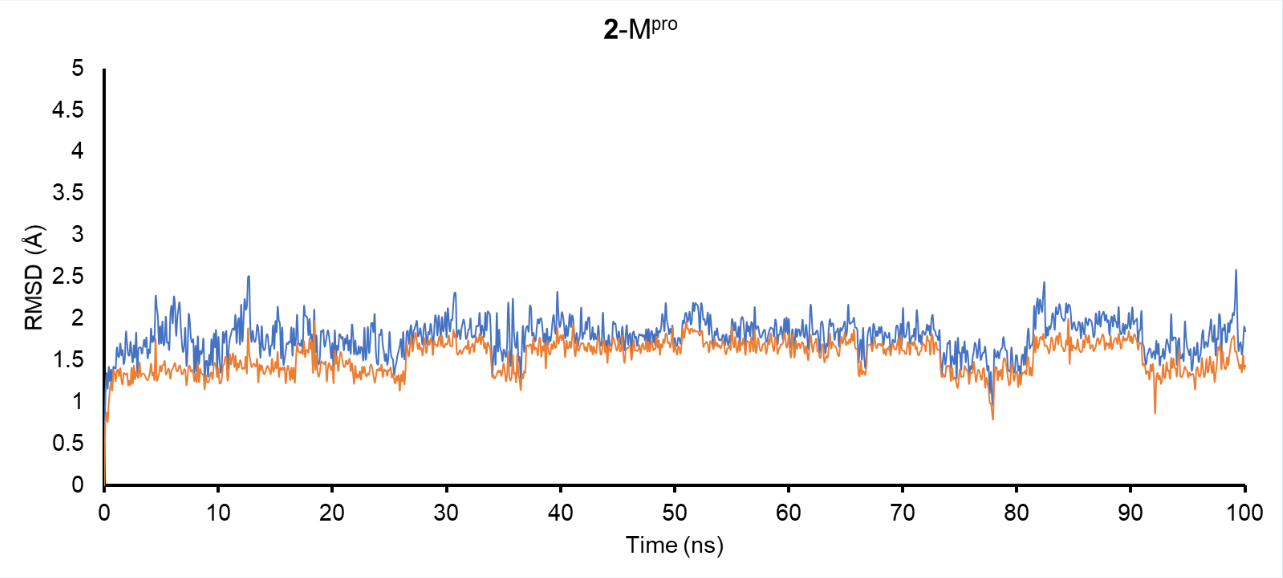

Figure S3. Heavy atom-positional RMSD of **2** respect to itself (orange line) and respect to the protein backbone (blue line) as a function of simulation time (ns). The latter RMSD is calculated by first aligning the protein-ligand complex on the protein backbone of the reference and then the RMSD of the ligand heavy atoms is measured.

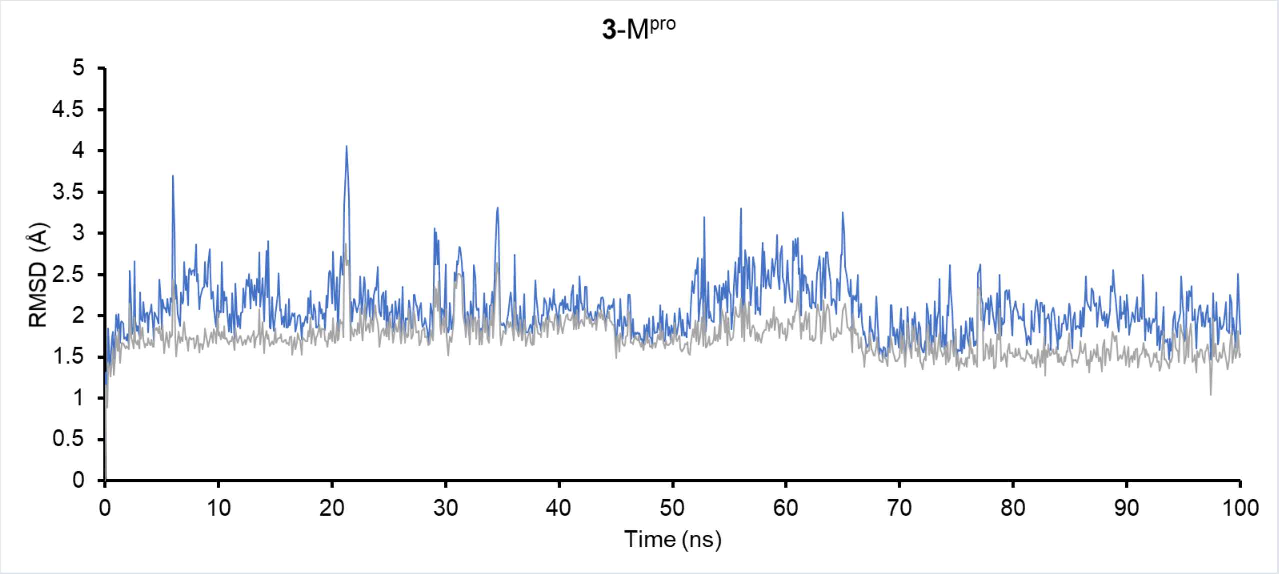

Figure S4. Heavy atom-positional RMSD of **3** respect to itself (grey line) and respect to the protein backbone (light-blue line) as a function of simulation time (ns). The latter RMSD is calculated by first aligning the protein-ligand complex on the protein backbone of the reference and then the RMSD of the ligand heavy atoms is measured.

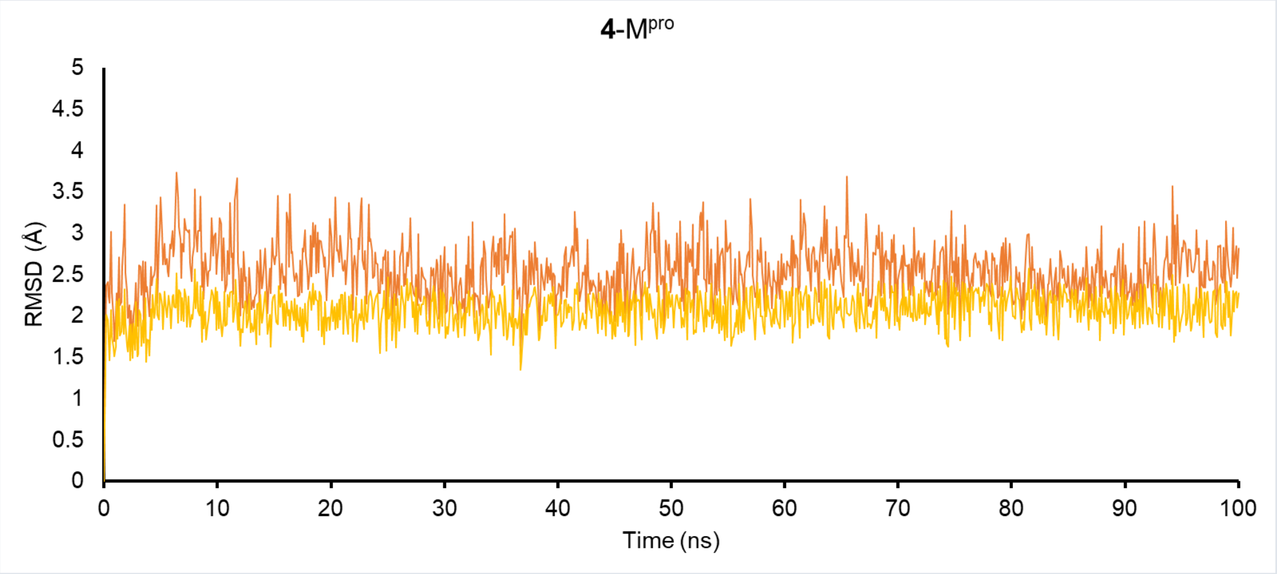

Figure S5. Heavy atom-positional RMSD of **4** respect to itself (yellow line) and respect to the protein backbone (orange line) as a function of simulation time (ns). The latter RMSD is calculated by first aligning the protein-ligand complex on the protein backbone of the reference and then the RMSD of the ligand heavy atoms is measured.

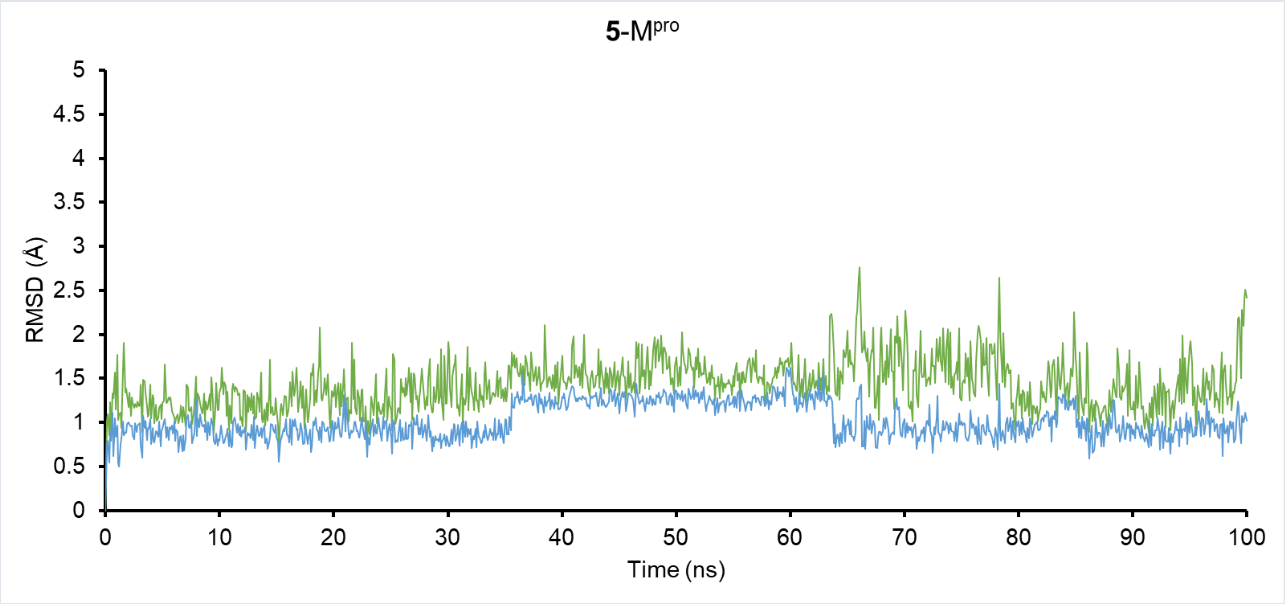

Figure S6. Heavy atom-positional RMSD of **5** respect to itself (blue line) and respect to the protein backbone (green line) as a function of simulation time (ns). The RMSD is calculated by first aligning the protein-ligand complex on the protein backbone of the reference and then the RMSD of the ligand heavy atoms is measured.

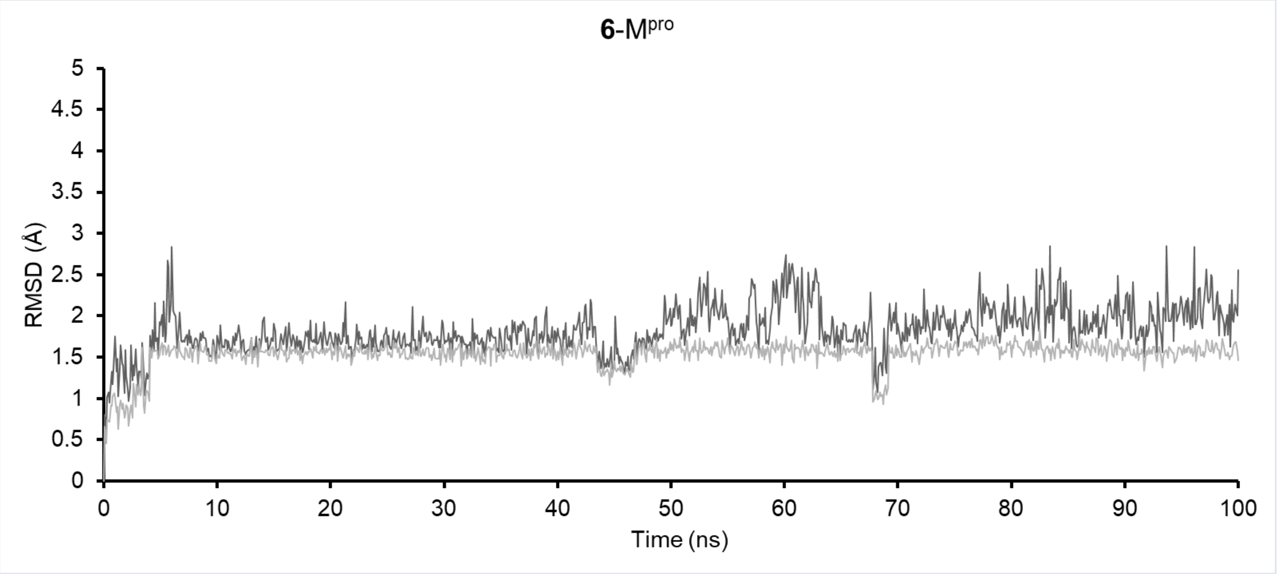

Figure S7. Heavy atom-positional RMSD of **6** respect to itself (light-grey line) and respect to the protein backbone (dark-grey line) as a function of simulation time (ns). The RMSD is calculated by first aligning the protein-ligand complex on the protein backbone of the reference and then the RMSD of the ligand heavy atoms is measured.

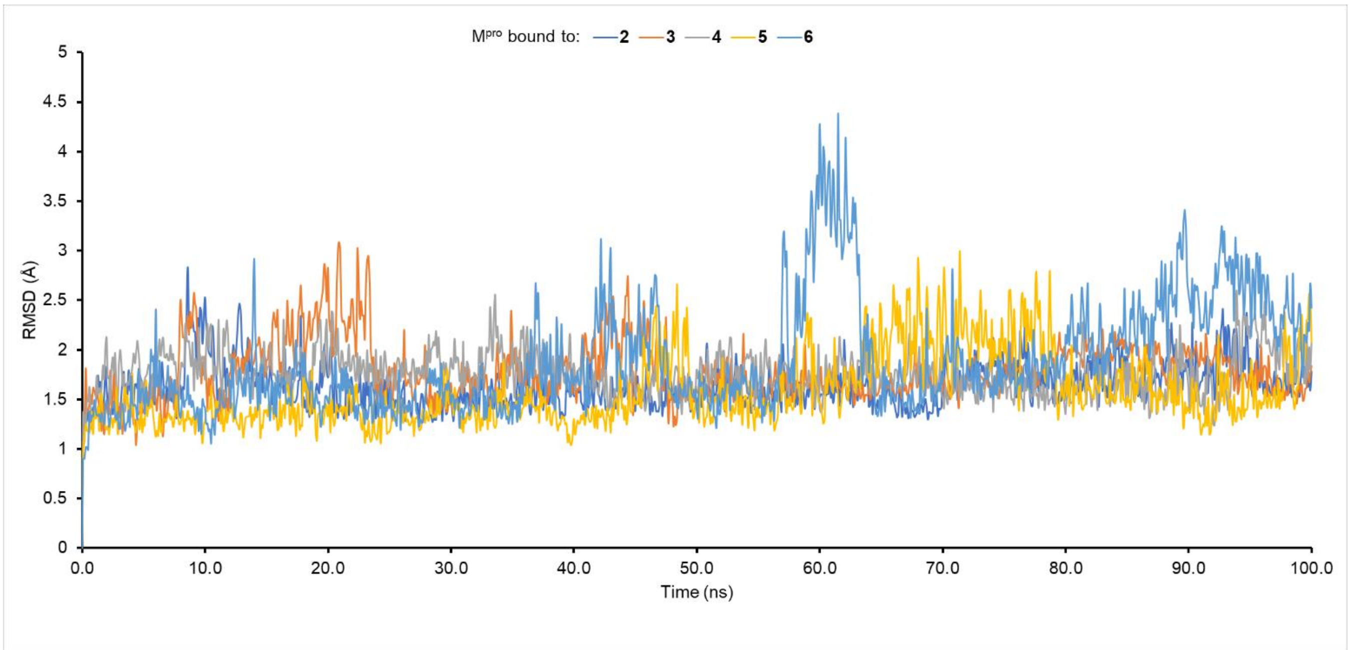

Figure S8. RMSD of protein Cα atoms (bound to **2**-**6**) as a function of simulation time (ns).

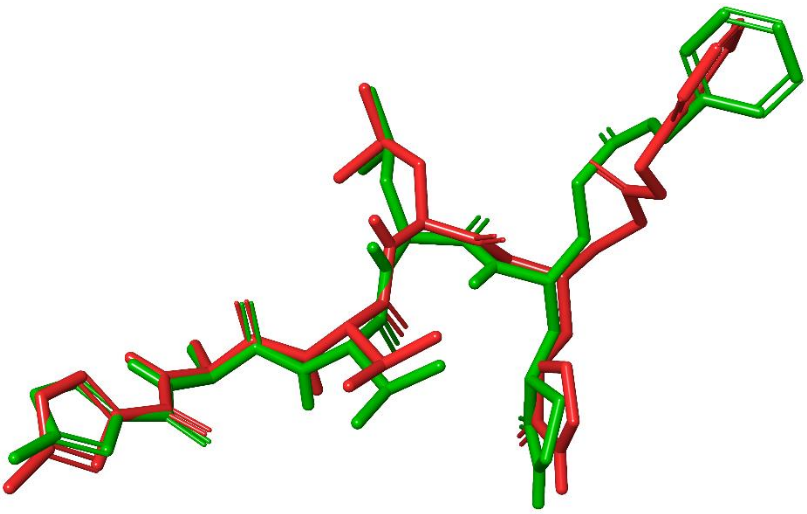

Figure S9. Superimposition (1.076 Å) of co-crystallized (red, PDB ID: 6LU7) and docked pose (green) structures of N3.

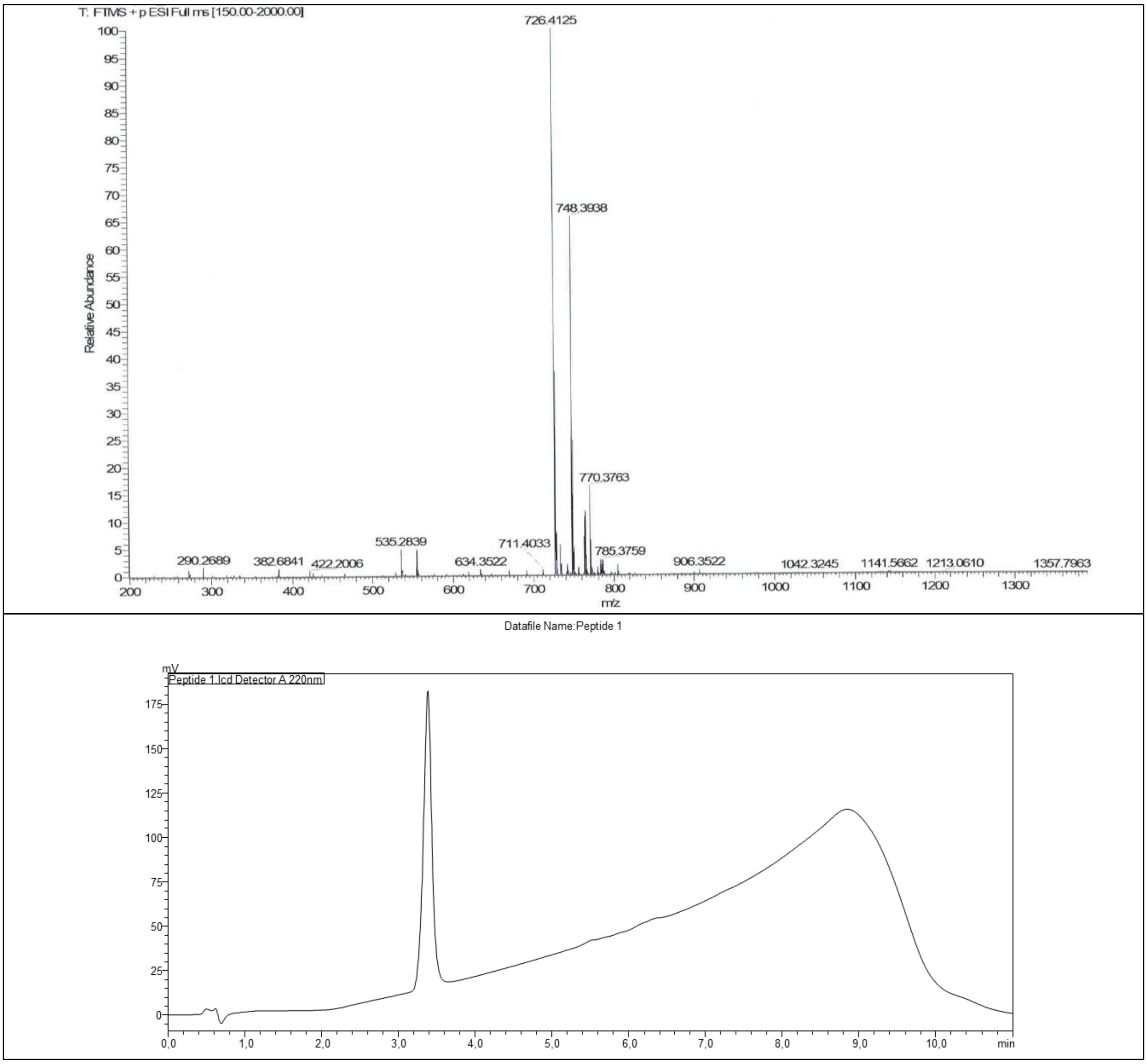

Figure S10. HR-ESI-MS of Peptide **1** ion $[M+H]^+$ (top) and analytical HPLC trace at 220 nm (bottom).

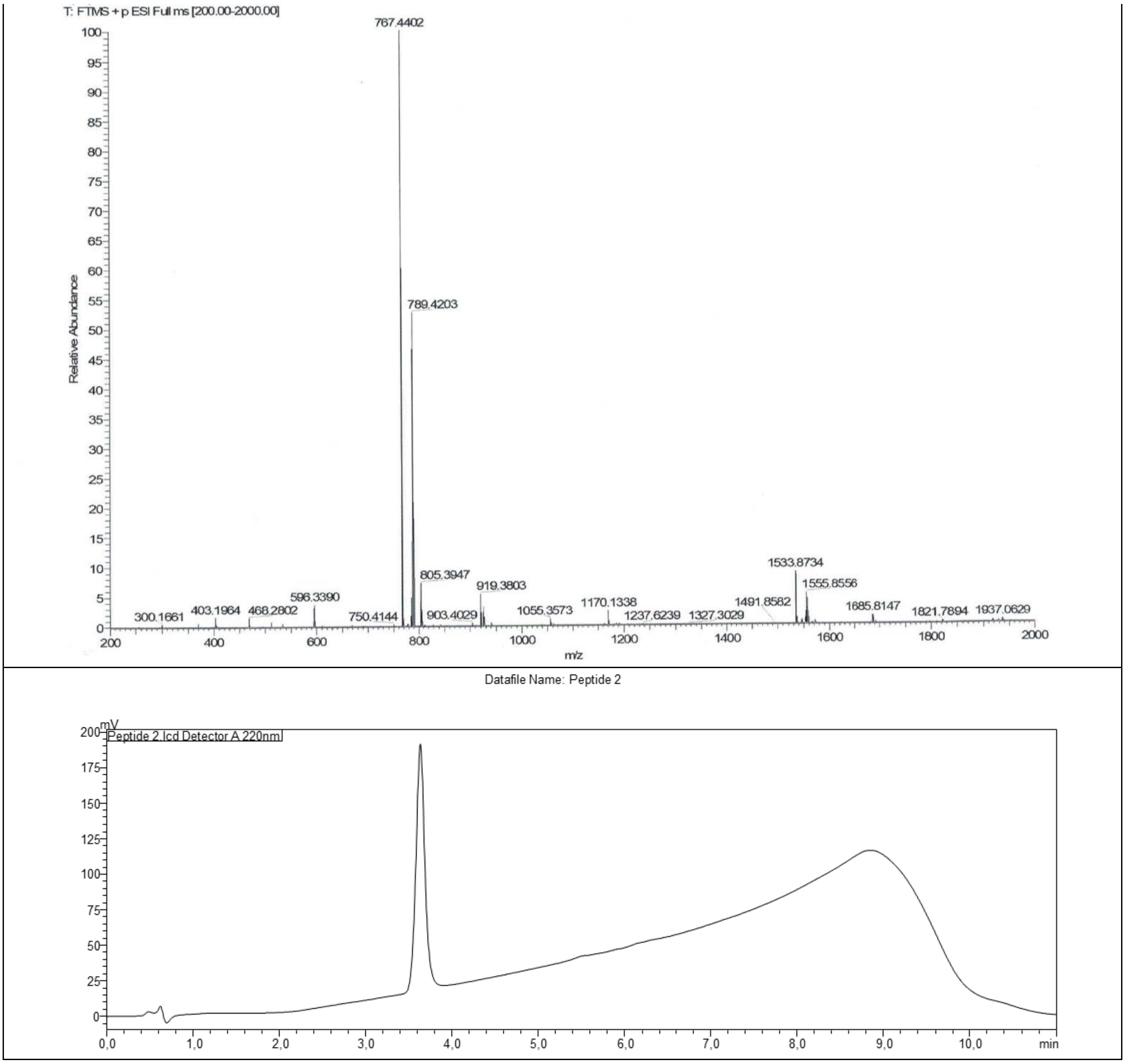

Figure S11. HR-ESI-MS of Peptide **2** ion $[M+H]^+$ (top) and analytical HPLC trace at 220 nm (bottom).

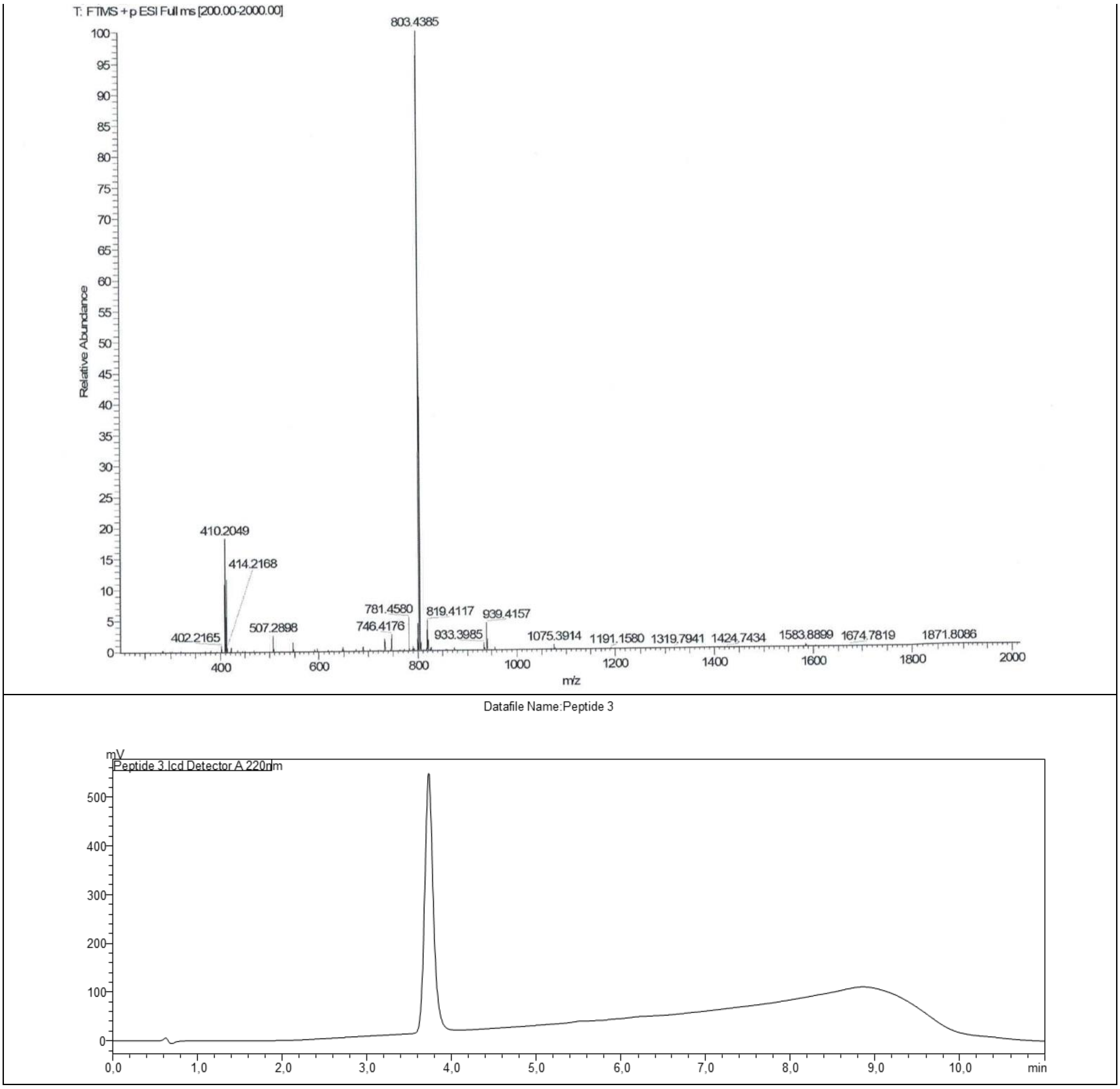

Figure S12. HR-ESI-MS of Peptide **3** ion $[M+H]^+$ (top) and analytical HPLC trace at 220 nm (bottom).

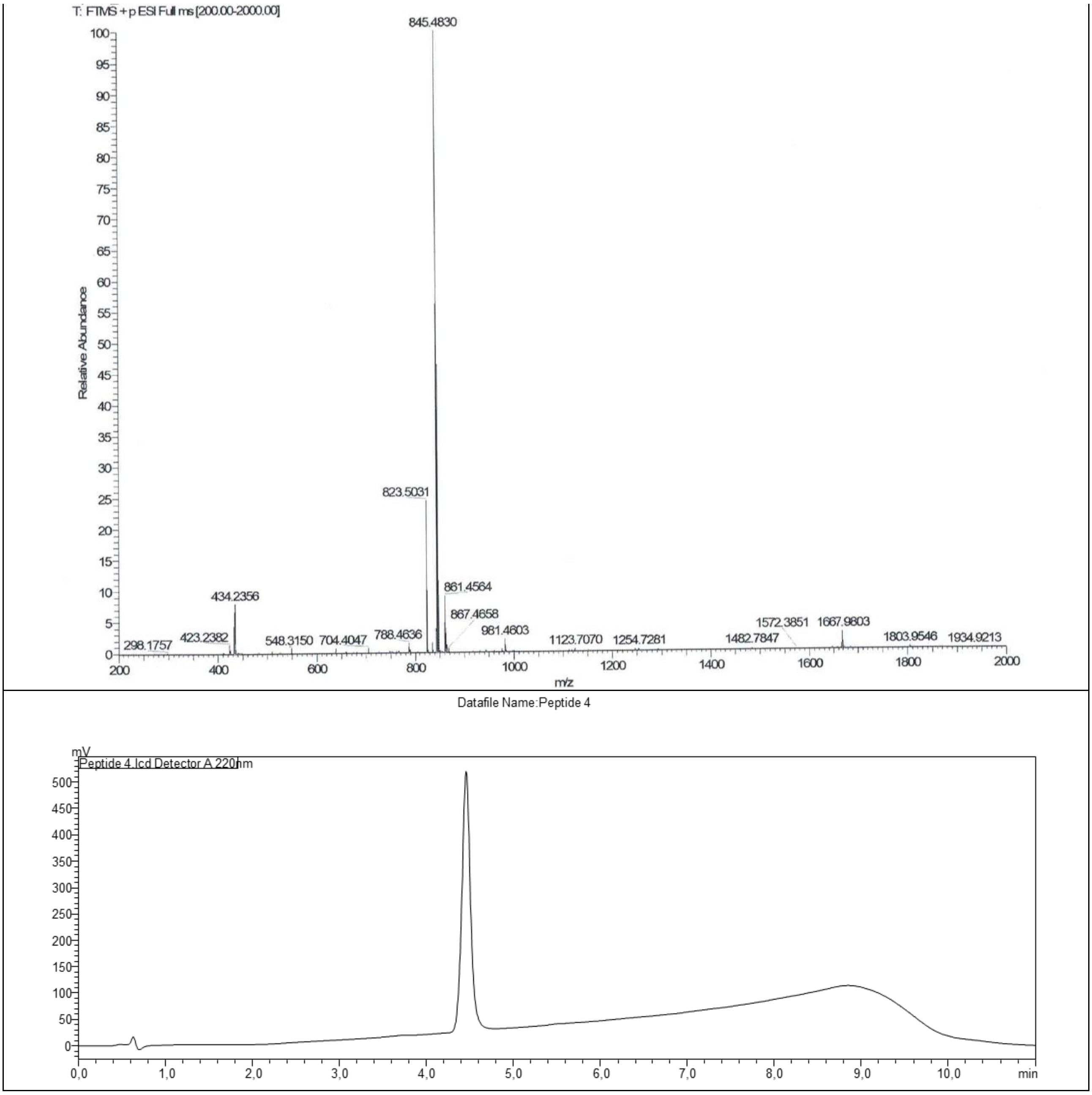

Figure S13. HR-ESI-MS of Peptide **4** ion $[M+H]^+$ (top) and analytical HPLC trace at 220 nm (bottom).

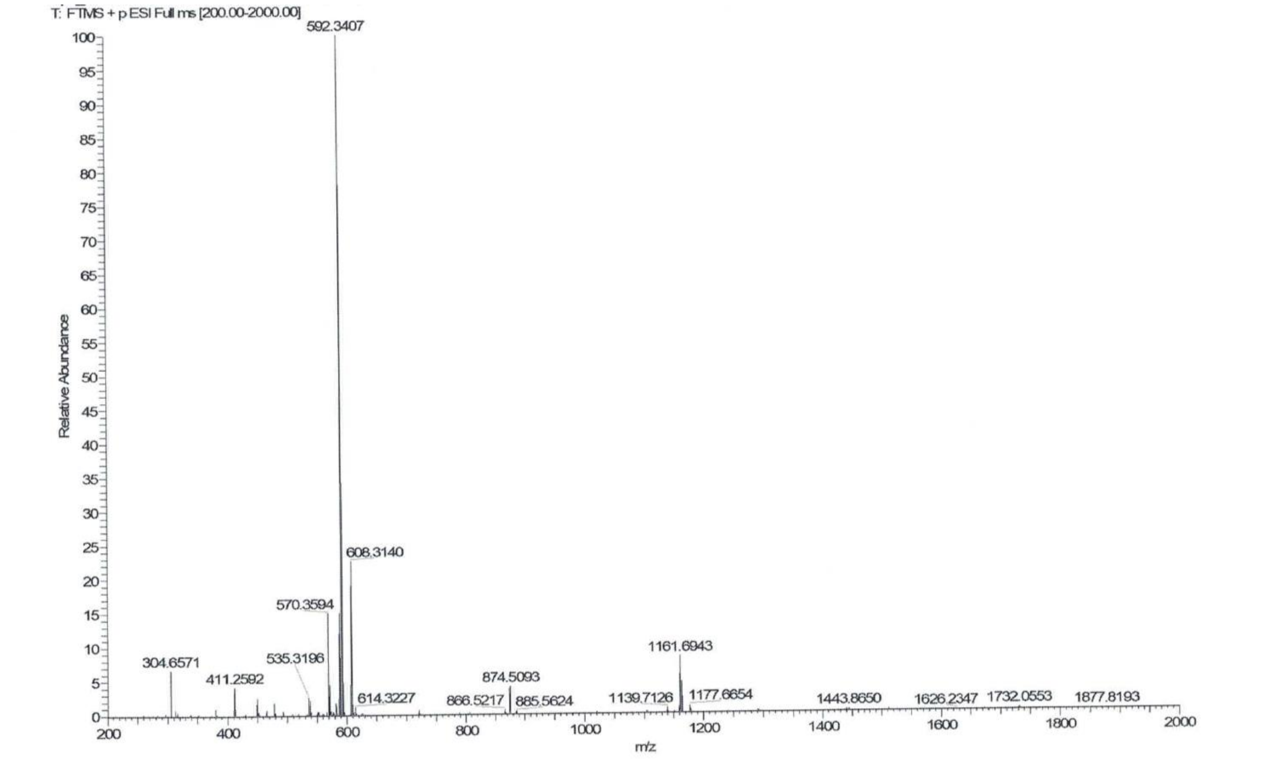

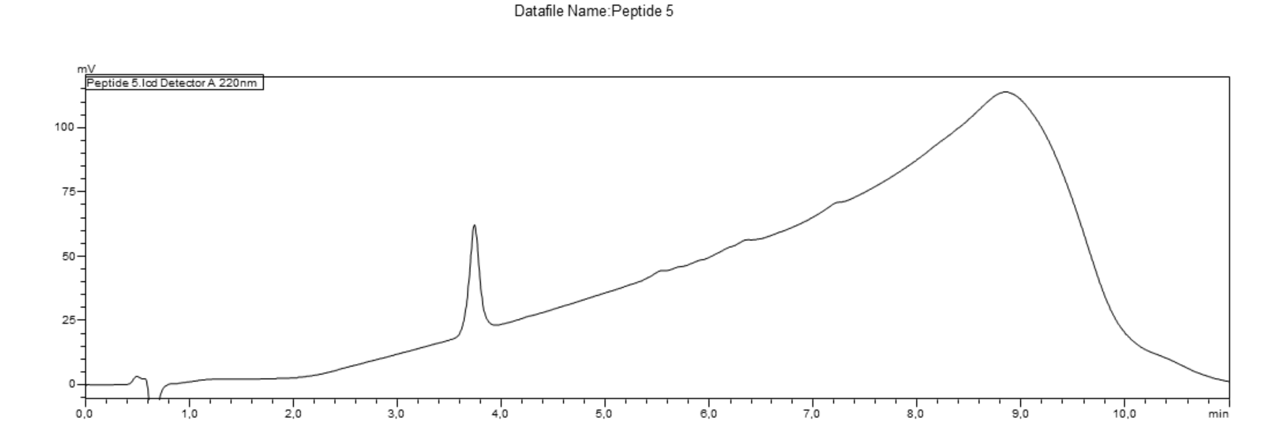

Figure S14. HR-ESI-MS of Peptide **5** ion $[M+H]^+$ (top) and analytical HPLC trace at 220 nm (bottom).

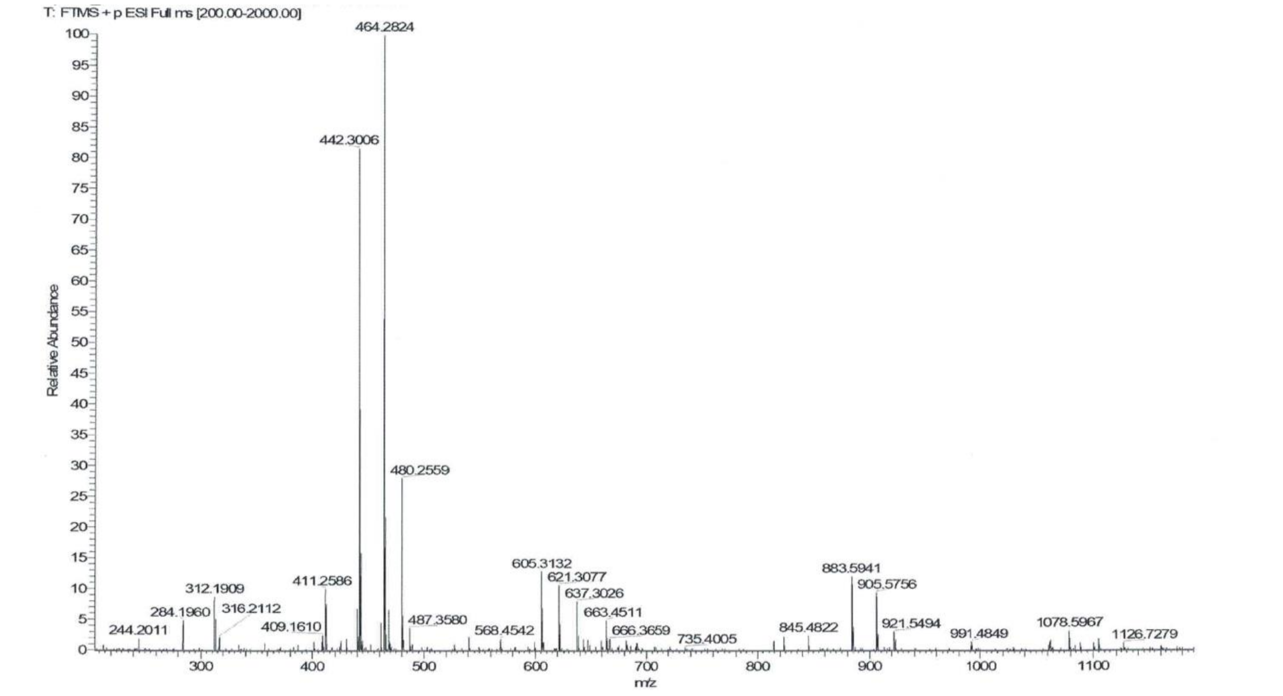

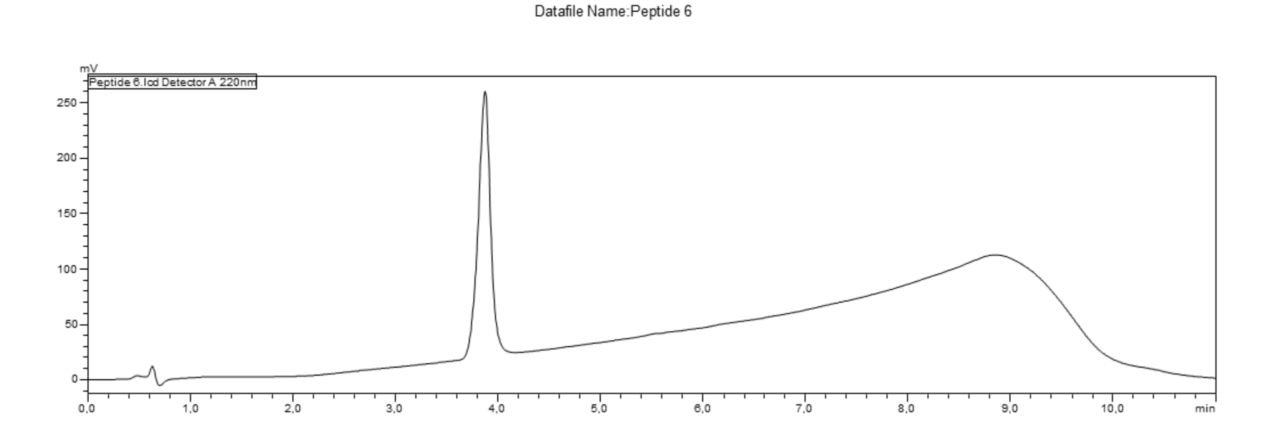

Figure S15. HR-ESI-MS of Peptide **6** ion $[M+H]^+$ (top) and analytical HPLC trace at 220 nm (bottom).

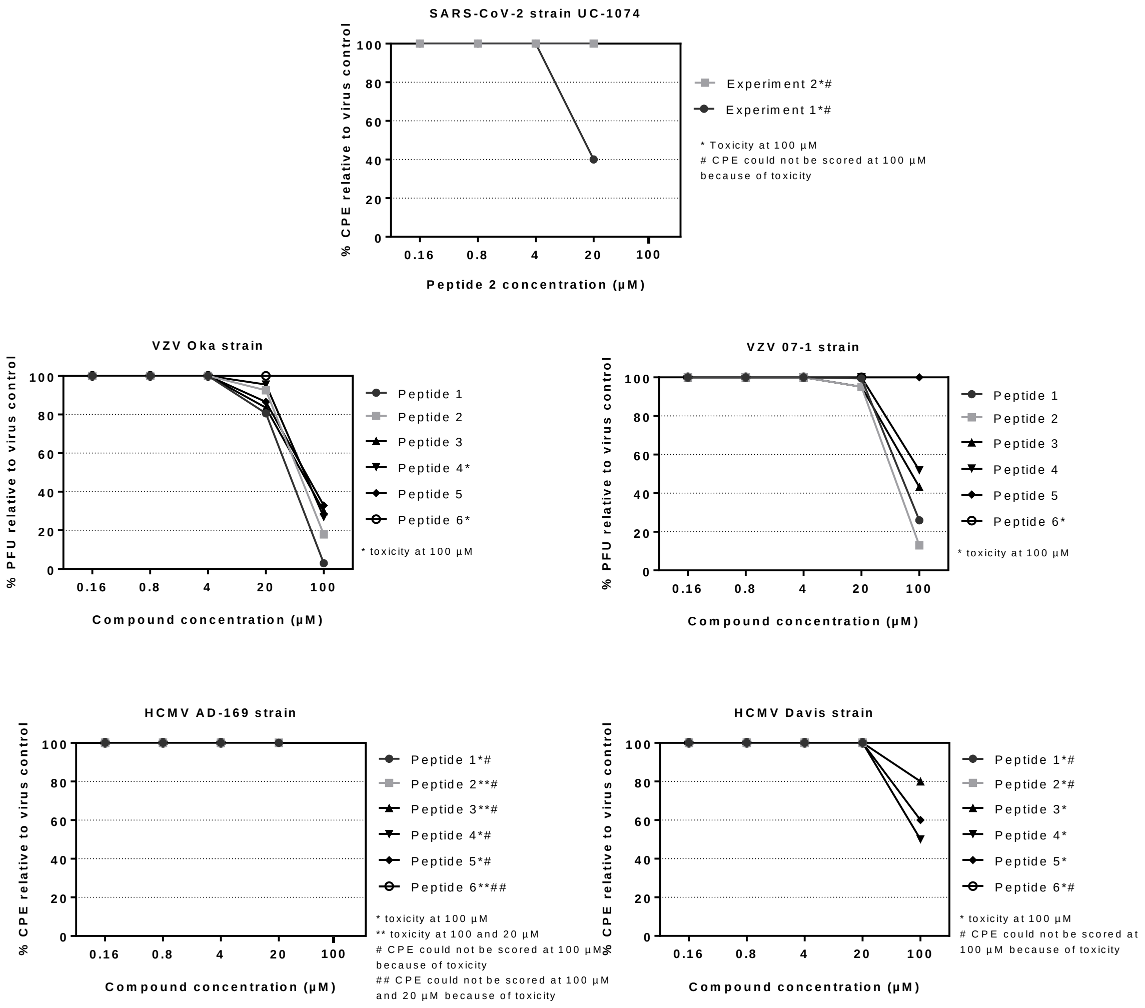

Figure S16. Representative dose-response curves.
